# Two-motif model illuminates DICER cleavage preferences

**DOI:** 10.1093/nar/gkad1186

**Published:** 2024-01-03

**Authors:** Cong Truc Le, Trung Duc Nguyen, Tuan Anh Nguyen

**Affiliations:** Division of Life Science, The Hong Kong University of Science & Technology, Hong Kong, China; Division of Life Science, The Hong Kong University of Science & Technology, Hong Kong, China; Division of Life Science, The Hong Kong University of Science & Technology, Hong Kong, China

## Abstract

In humans, DICER is a key regulator of gene expression through its production of miRNAs and siRNAs by processing miRNA precursors (pre-miRNAs), short-hairpin RNAs (shRNAs), and long double-stranded RNAs (dsRNAs). To advance our understanding of this process, we employed high-throughput dicing assays using various shRNA variants and both wild-type and mutant DICER. Our analysis revealed that DICER predominantly cleaves shRNAs at two positions, specifically at 21 (DC21) and 22 (DC22) nucleotides from their 5′-end. Our investigation identified two different motifs, mWCU and YCR, that determine whether DICER cleaves at DC21 or DC22, depending on their locations in shRNAs/pre-miRNAs. These motifs can work together or independently to determine the cleavage sites of DICER. Furthermore, our findings indicate that dsRNA-binding domain (dsRBD) of DICER enhances its cleavage, and mWCU strengthens the interaction between dsRBD and RNA, leading to an even greater enhancement of the cleavage. Conversely, YCR functions independently of dsRBD. Our study proposes a two-motif model that sheds light on the intricate regulatory mechanisms involved in gene expression by elucidating how DICER recognizes its substrates, providing valuable insights into this critical biological process.

## Graphical Abstract

**Graphical Abstract:**
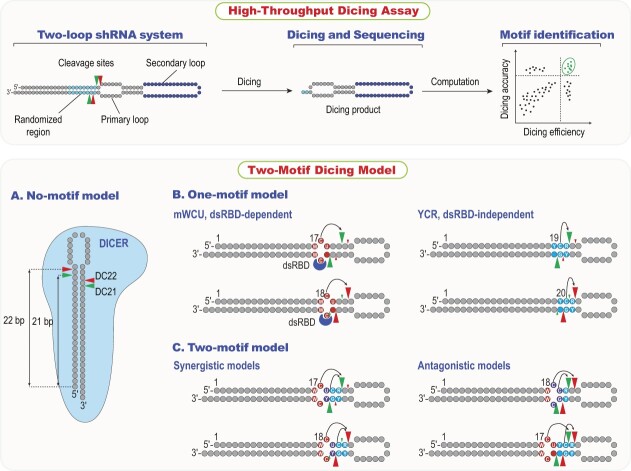

## Introduction

DICER is an essential RNase III enzyme involved in RNA silencing, cleaving pre-miRNAs into small dsRNAs of 21–25 base pairs (bp) (1,2). During animal canonical miRNA biogenesis, Microprocessor generates pre-miRNAs from primary miRNAs (pri-miRNAs) containing hairpin, which are further cleaved by DICER to generate small dsRNAs. Argonaute (AGO) binds to these dsRNAs, and one strand of each dsRNA is retained in AGO to form the core of the RNA-induced silencing complex (RISC). RISC then regulates gene expression through mechanisms such as mRNA cleavage, degradation, and/or translational inhibition (3,4). In addition to canonical miRNA biogenesis, many miRNAs are produced in the noncanonical miRNA biogenesis pathways, which do not require either Microprocessor or DICER (2,5). Proper activity of DICER and Microprocessor is critical for determining miRNA sequence and expression levels, and therefore, for normal functions of miRNAs. In addition, DICER cleaves short-hairpin RNAs (shRNAs) to generate siRNAs, which knock down gene expression. DICER cleavage is therefore also essential for shRNA technology (6–11). Moreover, DICER cleaves long dsRNAs, playing important roles in various cellular functions (1).

DICER acts as a ‘dsRNA molecular ruler’ in humans, measuring 21–22 nucleotides (nt) from the 5′- and 3′-ends of pre-miRNAs using two ‘RNA-binding pockets’ (12–21). DICER cleavage sites are influenced by various RNA elements, including the 5′- and 3′-ends of shRNAs/pre-miRNAs and positions of the apical loop (22–25). Our recent study utilized a two-loop shRNA system to investigate DICER cleavage efficiency and sites simultaneously (26). In this system, the primary loop serves as the interaction site for DICER, while the secondary loop contains randomized barcodes that allow us to map cleavage products to substrates, facilitating the identification of DICER cleavage sites. Using this approach, we discovered that the 22-bulge RNA element, located 22 nt from the 5′-end of shRNAs/pre-miRNAs, stimulates DICER to cleave at a position 2 nt away. Long-stem shRNAs/pre-miRNAs are preferentially cleaved at DC22, 22 nt from their ends, while short-stem RNAs are predominantly cleaved at DC21, 21 nt from their ends. Another recent study found that the ‘GYM’ motif can control DICER cleavage sites. The GYM motif, located 4 nt from the DICER cleavage site, consists of a C-G pair (represented by the ‘G’ in GYM), an A-U or G-C pair (referred to as the ‘Y’ component in GYM), and a mismatch containing C or A (known as the ‘M’ component in GYM) (27). In addition to RNA elements, TRBP, a co-factor of DICER, and the helicase domain of DICER have been shown to impact the cleavage sites of DICER in certain pre-miRNAs (28–34).

In our previous study, we demonstrated that by utilizing a two-loop shRNA system with a randomized region in and near the loop, we could identify RNA elements controlling DICER cleavage sites (26). These findings were verified in one-loop shRNAs and pre-miRNAs. Therefore, in this study, we employed high-throughput dicing assays using approximately 23,000 two-loop shRNAs containing randomized regions within the stem to further investigate DICER cleavage sites. Through this approach, we discovered two RNA elements that control the DICER cleavage, namely mWCU and YCR. We further demonstrated that mWCU is dependent on dsRBD, while YCR is not. Our proposed model suggests that DICER selects its cleavage sites based on the presence of these two motifs, either in a coordinated or uncoordinated manner. Our findings enhance our understanding of DICER mechanisms and their role in miRNA biogenesis.

## Materials and methods

### Human DICER expression and purification

The pXG-DICER and pXG-DICER△dsRBD plasmids are the same as those used in our previous study (26). pXG-DICER-R1855A and pXG-DICER-E1859A were generated from pXG-DICER using In-fusion cloning method (Supplementary Table S1). To express DICER (or DICER△dsRBD, DICER-R1855A, DICER-E1859A), pXG-DICER (or pXG-DICER△dsRBD, pXG-DICER-R1855A, pXG-DICER-E1859A) was transfected into 100 of 100 mm dishes of HEK293E cells, and the transfected cells were collected 3-day post-transfection. The cell pellets were resuspended in T500 buffer containing 20 mM Tris-HCl (pH 7.5), 500 mM NaCl, 4 mM β-mercaptoethanol (Thermo Fisher Scientific), 0.1 mg/mL RNase A (Thermo Fisher Scientific), and a protease inhibitor cocktail (Thermo Fisher Scientific). The resuspended cells were sonicated and subjected to high-speed centrifugation to obtain a 45 mL clear cell lysate, which was then mixed with 2 ml of Ni-NTA resin (Bio-Rad). The protein-bound resin was washed sequentially with three buffers containing 20 mM Tris-HCl (pH 7.5), 4 mM β-mercaptoethanol, and 2,000 mM NaCl (T2000), 0 mM NaCl (T0), or 500 mM NaCl (T500) supplemented with 40 mM imidazole. The resin-bound proteins were eluted from the Ni-NTA resin using T150 (20 mM Tris-HCl (pH 7.5), 4 mM β-mercaptoethanol, and 150 mM NaCl) plus 200 mM imidazole. The eluted proteins were then loaded onto Q Sepharose Fast Flow (GE Healthcare). The Q Sepharose beads were washed with T150, and the proteins were finally eluted from the Q Sepharose beads using T500-plus buffer containing 20 mM Tris-HCl (pH 7.5), 500 mM NaCl, 10% glycerol and 2 mM dithiothreitol (DTT) (Sigma-Aldrich).

### High-throughput shRNA cleavage assays

#### Randomized shRNA synthesis

Detailed information regarding all oligos utilized in this section can be referenced in Supplementary Table S2. Six RNA groups were generated using six single-stranded DNA (ssDNA) oligos with randomized nt sourced from Integrated DNA Technologies (IDT). Each ssDNA features a 32-nt random barcode region, a shRNA-encoding region containing two parts with a collective total of 6 random nt, and a 23-nt region complementary to the R-set6 primer (CTG AAG TAT CGG AAT ATG CAT GG). To synthesize a double-stranded DNA (dsDNA), 100 pmol of each oligo were annealed with 100 pmol of the R-set6 primer, forming a partial dsDNA with 23 bp in a 10 μL solution of 100 mM NaCl. This mixture was heated at 98°C for 3 min, then incubated at 65°C for 5 min, and finally chilled on ice for 1 min. Five units of Klenow fragment exo– (from Thermo Scientific) were employed to elongate the annealed R-set6 in a 20 μL reaction mixture at 37°C for 120 min, yielding a complete dsDNA. The resulting dsDNA was further amplified using F-T7 (TAA TAC GAC TCA CTA TAG GG) and R-set6 to obtain dsDNA containing the T7 promoter. Next, PsiI restriction enzyme (Thermo Scientific) was used to digest 500–1,000 ng of T7-containing dsDNA at 37°C for 120 min. The PsiI-digested dsDNA was then used in a 20 μl *in vitro* transcription reaction with the MEGAscript T7 transcription kit (Invitrogen) to synthesize RNA substrates. The IVT-synthesized RNA substrates were gel-purified, quantified using a NanoDrop 2000 spectrophotometer (Thermo Scientific), and stored at -80°C for later use.

#### The high-throughput dicing assays

For the high-throughput dicing (shRNA cleavage) assays, we incubated five pmol of each shRNA group (from 1 to 6) with four pmol of purified DICER, DICER△dsRBD, DICER-R1855A or DICER-E1859A in a 10 μl cleavage reaction buffer consisting of 50 mM Tris-HCl (pH 7.5), 150 mM NaCl, 10% glycerol, 0.2 μg/μL BSA, 1 mM DTT, and 2 mM MgCl_2_ at 37°C for 120 min. To stop the reaction, we added 10 μl of 2X-TBE buffer containing 2 mM Tris-HCl (pH 7.5), 20 mM EDTA (pH 8.0), and 8 M urea, and then incubated the resulting mixtures with 20 μg of proteinase K (Thermo Fisher Scientific) at 37°C for 15 min, and 50°C for 15 min. After heating at 95°C for 5 min, we analyzed the samples on a 12% urea-PAGE gel. The cleavage resulted in double cleavage (DC) and single cleavage (SC) fragments which were separately gel-purified.

#### The RNA cloning and sequencing for high-throughput dicing assays

We first ligated the OS (original substrate) and SC products to the 4N-RA3 adapter using T4 RNA Ligase 2, truncated KQ enzyme (NEB, M0373L). The resulting 4N-RA3-ligated OS and SC were then gel-purified. Next, we mixed the purified RNAs in a reverse transcription mixture containing cirRTP primer and Superscript IV Reverse Transcriptase (Invitrogen) and incubated the mixture for 60 min at 50°C. To degrade the RNAs, we added 0.1 M NaOH to the reverse transcription mixture and incubated it at 98°C for 10 min. We then purified the resulting cDNAs and circularized them using CircLigase ssDNA ligase (Epicentre). After separating the circularized cDNAs from linear cDNAs in an 18% urea-PAGE gel and gel-purifying them, we amplified the purified circularized cDNAs of OS or SC by PCR using RP1 and one of the RPIx primers, respectively. We used the RP1 and RPIx primer systems from the Truseq Illumina primers.

For the DC fragments, we ligated them to the 4N-RA3 adapter and separated the resulting 4N-RA3-ligated DC from unligated DC and free 4N-RA3 in a 12% urea-PAGE gel, which were then gel-purified. We ligated the purified 4N-RA3-ligated DC with the 4N-RA5 primer using T4 RNA ligase 1, and reverse-transcribed the double-ligated DC using Superscript IV Reverse Transcriptase and R-RA3 primer. Finally, we amplified the resulting cDNA by PCR using RP1 and RPIx. The concentration of the DNA libraries was measured by Qubit™ dsDNA HS Assay Kit.

In total, we generated at least two repeats for each DNA library of the high-throughput dicing assays for one enzyme, and we sequenced all libraries using Illumina NovaSeq 6000 in 150 bp paired-end mode.

The oligo sequences employed in this section can be found in Supplementary Table S3.

### Analysis of high-throughput dicing assays

To analyze the sequencing data, we followed the method previously described (26). First, we used cutadapt (-a TGGAATTCTCGGGTGCCAAGG -A GATCGTCGGACTGTAGAACTCTGAAC -m 10) to remove the adapters from the raw reads (35). Next, we joined the pair-end reads using fastq-join (36), and then filtered out the low-quality reads using fastq_quality_filter (-q 20 -p 90). We removed any duplicated reads containing the same 4 nt or 6 nt randomized barcodes in both ends using fastx_collapser (http://hannonlab.cshl.edu/fastx_toolkit/index.html, version 0.0.13). We then processed the OS, DC and SC samples separately.

For the OS libraries, we used cutadapt (cutadapt -u 6 -u -4) to remove the 6-nt randomized barcodes in the 5′-end and 4-nt randomized barcodes in the 3′-end of the OS reads. We split each resulting read into two segments (FL-OS and 32N) using cutadapt (cutadapt -g GCTTGC…GCAAGC -m 32 -M 32 –discard-untrimmed) (35). We discarded any FL-OS/32N pairs that contained the 32N barcode shared by two or more FL-OS, obtaining the unique FL-OS/32N dictionary. We aligned the resulting FL-OS sequences with the reference sequences containing 23,296 possible variants of 6 shRNA groups using BWA (37). We selected only the perfectly aligned FL-OS sequences for further analysis. The raw counts of each FL-OS were the sum of read counts of the FL-OS in the unique FL-OS/32N dictionary.

On the other hand, we used cutadapt to remove randomized barcodes in both ends of DC or SC reads (cutadapt -u 4 -u -4: 4 nt in both ends for DC reads; cutadapt -u 6 -u -4: 6 nt in 5′-end and 4 nt in 3′-end for SC reads) (35). We then split the processed reads into two fragments, including a cleaved shRNA product (CP) and a 32N barcode, using cutadapt (cutadapt -g GCTTGC…GCAAGC -m 32 -M 32 –discard-untrimmed). For each pair of CP/32N, we used the 32N barcode sequence to assign the CP to the FL-OS sequence in a pair with the barcode in the FL-OS/32N dictionary. We determined the cleavage sites by mapping the CP and FL-OS using the local alignment mode of pairwise2 from Biopython (38). We labeled the modes of DICER cleavage as 5′-SC (single cleavage on 5′-strand), 3′-SC (single cleavage on 3′-strand) or DC (double cleavage) based on the reported mapping coordinates (*x*, *y*), where *x* and *y* are the 5′ and 3′ cleavage sites counting from the first nt of shRNA variants. We defined the cleavage modes as follows:

- Double cleavage with 2-nt overhang (DC) was performed at *x* if 19 ≤ *x* ≤ 23 and *y* = 72 – *x*;
- Other double cleavages (Other) with non-2-nt overhang were performed if 19 ≤ *x* ≤ 23 and 68 ≤ *y* ≤ 72 and *y* ≠ 72 – *x*;
- Single cleavage on 5′-strand (5′-SC) was performed at *x* if 19 ≤ *x* ≤ 23 and 68 ≤ *y* ≤ 72;
- Single cleavage on 3′-strand (3′-SC) was performed at 72 – *y* if 0 ≤ *x* ≤ 4 and 49 ≤ *y* ≤ 53.

For each variant, the raw read counts were normalized in each sample as the read counts per million. The cleavage efficiency and accuracy of DICER at each cleavage site were calculated separately for single cleavage and double cleavage.

The local cleavage efficiency score at the cleavage site P was calculated by log_2_(NP + 0.1) – log_2_(NS + 0.1).

The total cleavage efficiency for each variant was calculated separately for double cleavage and single cleavage by log_2_(∑NP + 0.1) – log_2_(NS + 0.1).

NP was the normalized count of the cleaved product at the cleavage site P; NS was the normalized count of the original substrate that generated this product. 0.1 was a pseudocount.

The cleavage accuracy score at the cleavage site P was calculated by NP/∑NP.

The average cleavage efficiency and cleavage accuracy values of each variant were obtained from three repeats.

Log_2_(DC/SC ratio) was calculated for each variant by log_2_(∑NP_DC_ + 0.1) – log_2_(∑NP_SC_ + 0.1), where NP_SC_ and NP_DC_ were the normalized count of the cleaved product at the cleavage site P for single cleavage and double cleavage products, respectively. 0.1 was a pseudocount. The average log_2_(DC/SC ratio) value was estimated for each variant for WT and mutant DICER from three repeats.

RNAfold (ViennaRNA Package version 2.4.9) was used to predict the secondary structure of each variant, using the default parameters (39). The dot-bracket structures obtained from RNAfold were then converted into our custom format, where each position was annotated with one of six features: L (loop), b (base pair), M (symmetric mismatch), A (asymmetric mismatch), B (bulge), and T (3′-overhang). We selected 36 structures from the shRNA library that contained at least 50 variants from the full library, which included a total of 22,512 out of 23,296 shRNA variants. To determine the length of the stem, we counted the number of bp and symmetric mismatches on the 5′-strand from the first bp of the stem to the apical loop.

### Quantification of mWCU and YCR scores

The cleavage accuracy scores of DICER at DC20, DC21 and DC22 for shRNA variants with randomized nt in positions 16–18, 17–19 and 18–20 were rescaled from 0 to 100. These values were named mWCU-DC20, mWCU-DC21 and mWCU-DC22, respectively. As a result, each motif received a score ranging from 0 to 100 using the min-max normalization method. The average accuracy scores of these motifs were calculated as the mean of mWCU-DC20, mWCU-DC21 and mWCU-DC22 scores, and were referred to as mWCU scores. List of mWCU scores is presented in Supplementary Table S4.

For scoring YCR motifs, which are independent of dsRBD, we used DICERΔdsRBD sequencing data to eliminate the effect of dsRBD. The cleavage accuracy scores at DC20, DC21 and DC22 for shRNA variants with randomized nt in positions 18–20, 19–21 and 20–22 were rescaled from 0 to 100, resulting in YCR-DC20, YCR-DC21 and YCR-DC22 scores, respectively. Consequently, each motif received a score ranging from 0 to 100 using the min-max normalization method. The average accuracy scores of these motifs were calculated as the mean of YCR-DC20, YCR-DC21 and YCR-DC22 scores, and were referred to as YCR scores. In some cases, when YCR-DC22 could not be obtained, the YCR scores were calculated as the average of YCR-DC20 and YCR-DC21 values. List of YCR scores is presented in Supplementary Table S5.

It is important to note that we only selected shRNA variants with a consistent 22-bp stem length. Therefore, motifs that created different structures, altering the shRNA stem length, were excluded from the analysis. Ultimately, we obtained 3,851 mWCU motifs and 1,156 YCR motifs for calculating accuracy scores in each 3-bp randomized window, instead of the 4,096 motifs identified in each window by high-throughput dicing assays.

### Statistics

To compare the difference of two datasets, we calculated Cohen's *d* value, which quantifies the standardized difference between their mean values (40). This is computed using the formula *d* = (M1 – M2)/pooled_SD, where M1 and M2 represent the means of dataset 1 and dataset 2, respectively. The pooled_SD value is calculated as √(SD1^2^/2 + SD2^2^/2), where SD1 and SD2 are the standard deviations of dataset 1 and dataset 2, respectively.

### shRNA synthesis

Comprehensive details pertaining to all oligos used in this section can be found in Supplementary Table S6. To synthesize each shRNA, an ssDNA containing a sequence complementary to the shRNA and a sequence complementary to the T7 promoter was initially purchased. 100 pmol of each ssDNA were annealed with 100 pmol of the T7 primer (TAA TAC GAC TCA CTA TAG) in a 10 μL solution of 100 mM NaCl. The annealing program consisted of heating at 98°C for 3 min, incubating at 65°C for 5 min, and finally chilling on ice for 1 min. Five units of Klenow fragment exo– were added to this annealed dsDNA in a 20 μL reaction mixture and incubated at 37°C for 120 min, resulting in complete dsDNA. Subsequently, 500 ng of Klenow-synthesized dsDNAs were used in a 10 μL *in vitro* transcription reaction, which was carried out using the MEGAscript T7 transcription kit (Invitrogen). The resulting shRNA substrates were gel-purified, quantified using a NanoDrop 2000 spectrophotometer (Thermo Scientific), and stored at -80°C for later use.

### Pre-miRNA synthesis

Detailed information for all oligos utilized in this section is available in Supplementary Table S7. To synthesize human pre-miRNAs, we obtained pre-miRNA sequences from MirGeneDB (41). dsDNA templates of canonical (one-loop) pre-miRNAs containing a T7 promoter, a Hammerhead ribozyme sequence, and the full pre-miRNA sequence were synthesized from normal PCR using three ssDNAs as forward primer, reverse primer, and PCR template.

Each dsDNA utilized for creating a two-loop pre-miRNA was synthesized via an overlapping PCR reaction using two specific dsDNAs. Each of these two dsDNAs was synthesized in two steps. The first step involved annealing two synthetic ssDNAs using the following program: the mixture was heated at 98°C for 3 min, incubated at 65°C for 5 min, and finally chilled on ice for 1 min. In the second step, the annealed dsDNA was converted into a complete dsDNA by adding five units of Klenow fragment exo– in a 20 μl reaction mixture which was then incubated at 37°C for 120 min. Subsequently, the two dsDNAs underwent overlapping PCR reactions to synthesize a full-length dsDNA template. This template contained a T7 promoter, a Hammerhead ribozyme sequence, and the complete pre-miRNA sequence with a 32N secondary loop.

We used 500 ng of the resulting dsDNA in the *in vitro* transcription to synthesize RNAs. The synthesized RNAs were purified using phenol extraction and IPA precipitation and then treated with 40 mM MgCl_2_ at 72°C for 1 min, 65°C for 5 min and 37°C for 10 min for three cycles to activate the self-cleavage activity of the Hammerhead ribozyme, releasing the pre-miRNA sequences. The Hammerhead-cleaved RNAs were heat-denatured at 95°C for 5 min by adding 2X-TBE buffer containing 8 M urea, 20 mM EDTA and 2 mM Tris-HCl pH 7.5, and separated on a pre-run 10% urea-PAGE gel at 300 V. The cleaved pre-miRNAs were purified from the gel, which had 5′-OH ends. We converted this 5′-OH-containing pre-miRNAs into 5-monophosphate-containing pre-miRNAs using the T4 PNK enzyme and ATP. Finally, the 5-monophosphate pre-miRNAs were purified using isopropanol and stored at -80°C for future use.

### shRNA/pre-miRNA cleavage assays

Each cleavage assay reaction consisted of 10 μL reaction buffer containing 50 mM Tris-HCl (pH 7.5), 150 mM NaCl, 10% glycerol, 0.2 μg/μL BSA, 1 mM DTT and 2 mM MgCl_2_, with varying amounts of purified DICER as indicated in the figure legends. Three pmol of each RNA substrate were added to the reaction mixture. The reactions were incubated at 37°C for 120 min and then stopped by adding 10 μL of 2X-TBE buffer and 20 μg of Proteinase K (Thermo Fisher Scientific). The resulting mixture was incubated at 37°C for 15 min, followed by 50°C for 15 min, and then heated at 95°C for 5 min. The mixtures were subsequently analyzed by pre-run 12% urea-PAGE and stained with SYBR™ Green II RNA gel stain (Invitrogen).

### Reporter assays

To generate pGL-FL-a or pGL-FL-b plasmids, the binding sequence of siRNAs targeting gene A and gene B was inserted in the 3′-UTR of Firefly luciferase in pGL plasmid. shRNA plasmids were constructed using pU6-Sp-pegRNA-HEK3_CTT_ins (Addgene plasmid # 132778) as a backbone. HEK293T cells in 96-well plates were transfected with 150 ng pGL-FL plasmid, 25 ng of Renilla luciferase (RL) plasmid (pGL-RL), and varying amounts of shRNA plasmid using Lipofectamine 3000 (Thermo Fisher Scientific). After 24 h, the transfected cells were lysed in 20 μL of Promega E1980 lysis buffer, and Firefly and Renilla luciferase activities were measured using luciferase substrate and a multi-mode reader (flexstation 3 multi-mode microplate reader). The relative expression of Firefly luciferase was normalized to that of Renilla luciferase. Refer to Supplementary Table S8 for the sequences of oligos used in this section.

### Single nucleotide polymorphism analysis

We obtained 567 wild-type pre-miRNA (WT-pre-miRNA) sequences from MirGeneDB (41) and collected single nucleotide polymorphisms (SNPs) and mutations that occurred in these pre-miRNA sequences from miRNASNPs-v3 (42). We generated SNP-pre-miRNA sequences and focused on positions 17–22, which are potential positions for mWCU and YCR. Using RNAfold (39), we folded both the WT-pre-miRNA and SNP-pre-miRNA sequences, and then assigned the mWCU and YCR motif scores for each pre-miRNA. SNPs that either enhance or reduce the motif scores at least 20 units were selected. The sequences analyzed in this section are shown in Supplementary Tables S9 and S10.

### The motif analysis for pre-miRNAs

We collected pre-miRNA sequences from 34 animal species through MirGeneDB (41). For each pre-miRNA, we identified the DICER cleavage sites from the 5′-end of mature 3p miRNA or the 3′-end of mature 5p miRNA. We also used RNAfold (39) to fold the pre-mRNA sequences. We looked at the window from 1–5 nt and from 1–7 nt from the DICER cleavage site towards the 5′-end of the pre-mRNA sequences to examine the presence of YCR and mWCU, respectively. However, we excluded 113 members of the miR-430 family in *X. tropicalis* from our analysis. This is because their structural similarity caused bias in the enrichment analysis of the motifs. The sequences analyzed in this section are presented in Supplementary Tables S11 and S12.

### Rescue experiment and small RNA library construction

A rescue experiment was conducted on DICER knockout (DICER-KO) cells, derived from HCT116 cells. These KO cells were a generous gift from Dr. Narry Kim's lab at Seoul National University. Two μg of each plasmid (pXG-DICER, pXG-DICER△dsRBD, pXG-DICER-R1855A or pXG-DICER-E1859A) were transfected into 6-well plates containing cells at 50% confluency using Lipofectamine 3000 (Invitrogen, L3000075) in accordance with the manufacturer's protocol. Cells were harvested 48 h post-transfection, and total RNA was extracted using Trizol (Ambion, 15596018).

The construction of the small RNA library was performed using the NEBNext® Small RNA Library Prep Set for Illumina (NEB, E7330S), following the manufacturer's protocol with certain modifications. Briefly, small RNA fractions were separated and purified using 12% urea-PAGE gels. The purified RNAs were then ligated with a 3′-adapter. A blocking oligo was used to prevent the reaction of the excess adapter in subsequent steps. Next, a 5′-adapter was ligated to the 3′-adapter-ligated RNAs. The ligated small RNAs were reverse-transcribed, and the resulting cDNAs were amplified with index primers to generate the DNA libraries. All experiments were conducted in triplicate.

### Small RNA sequencing analysis

In addition to our small RNA sequencing libraries created in the rescue experiment of this study, we also incorporated small RNA sequencing data from a previous study (27). This data can be accessed in the GEO repository under the accession number GSE202535. The data were processed using established methods (27). We selected 3p miRNAs with unmodified ends and more than 10 raw reads, focusing on the analysis of the 5′-end of these miRNAs to identify DICER cleavage sites on pre-miRNAs. We classified the DICER cleavage sites as DCx, where x represents the length of the miRNA without considering bulges or asymmetric mismatches. Specifically, we further analyzed pre-miRNAs containing the DC21 miRNA isoforms, which accounted for more than 5% of all DCs. We estimated the fold changes in the proportion of DC21 between DICERΔdsRBD and DICER-WT to examine the effect of dsRBD on DC21 accuracy. The number of pre-miRNA harboring 17-mWCU motifs detected in HCT116 sequencing data was low as 2, so we excluded that sample and performed analysis only for HEK293T samples.

### Protein sequence and structure alignment

The sequences of animal DICER's dsRBD were sourced from Uniprot and aligned using Mutalin (43), with a consensus threshold set at 100%. The structures of human and mouse DICER's dsRBD were derived from experimentally solved apoprotein structures, specifically, 7XW3 and 7YZ4 respectively. In contrast, the structures of fruit fly and roundworm DICER's dsRBD were predicted using AlphaFold (Access the database at https://alphafold.ebi.ac.uk/), with the respective accession IDs being AF-Q9VCU9-F1 and AF-P34529-F1. Superimposition was executed using PyMOL (available at http://www.pymol.org).

## Results

### dsRBD facilitates DC21 cleavage of DICER

We purified DICER-WT and DICERΔdsRBD, and investigated their cleavage activity using pre-let-7a-1 substrate (Supplementary Figure S1A, B). As expected, DICER-WT produced three fragments upon cleaving this pre-miRNA. Similarly, DICERΔdsRBD also cleaved this pre-miRNA generating three similar fragments but exhibited more single cleavage events compared to DICER-WT (Supplementary Figure S1C), which is consistent with our previous study (26). These results suggest that both enzymes can be utilized for further research.

**Figure 1.**
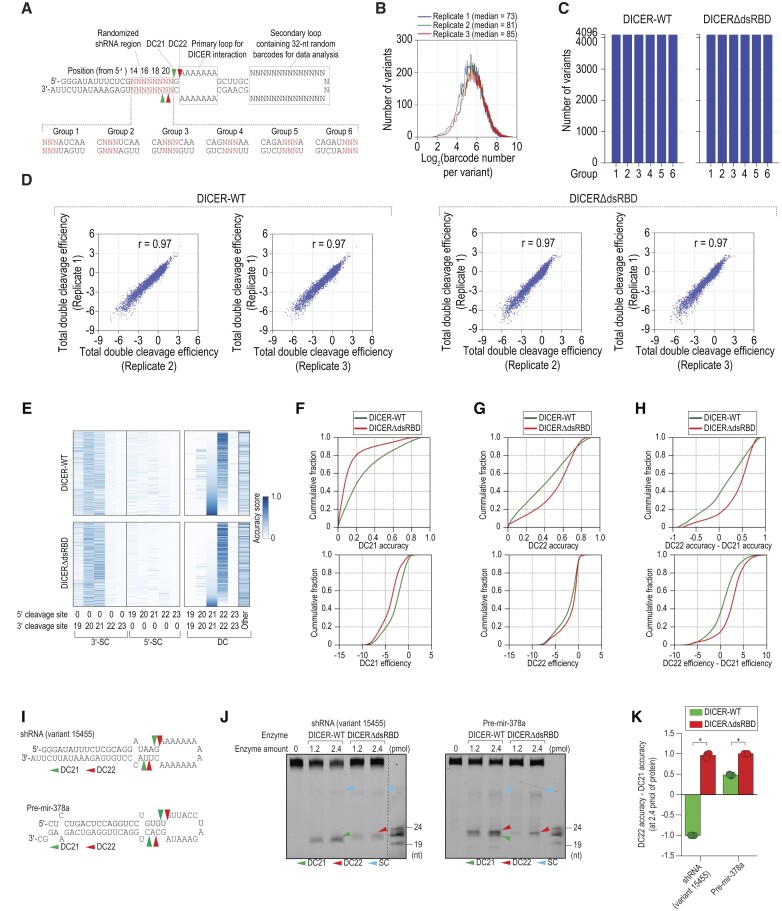
dsRBD facilitates DICER DC21 cleavage. (**A**) Schematic diagram of the two-loop shRNA containing primary and secondary loops. The stem region, located 14–21 nt from the 5′-end of shRNA, was randomized in 3-bp windows, generating 6 groups. The secondary loop contained 32-nt randomized sequences (32N) that served as barcodes. Green and red arrowheads indicate DICER's double cleavage (DC) at positions 21 and 22 from the 5′-end of shRNA, respectively. (**B**) Distribution of log_2_(barcode numbers) for shRNA variants identified in three repeated high-throughput dicing assays. (**C**) Identification of synthesized shRNA variants in the high-throughput dicing assays. Blue bars indicate the number of identified shRNA variants in each group. 4,096 represents all possible variants in each group. (**D**) Reproducibility of the three repeated high-throughput cleavage assays. The double cleavage efficiency was estimated for each variant in each repetition of the assays. Each dot in the plots represents one shRNA variant. R is Pearson's correlation coefficient. (**E**) DICER accuracy scores for each cleavage type at different positions: 5′-SC (single cleavage on 5′-strand), 3′-SC (single cleavage on 3′-strand), and DC (double cleavages). Accuracy scores for 5′-SC, 3′-SC, and DC of DICER at positions ranging from 19 to 23 in shRNAs were calculated using NP/∑NP, where NP represents the normalized count of the cleaved product at cleavage site P. Each line in the graph corresponds to one randomized shRNA variant in the library. (**F, G**) Cumulative plots showing the difference in DC21 or DC22 cleavage accuracy (top panel) and efficiency (bottom panel) between DICER-WT and DICERΔdsRBD. (**H**) Cumulative plot comparing the difference in DC22 and DC21 cleavage accuracy (top panel) and efficiency (bottom panel) between DICER-WT and DICERΔdsRBD. (**I**) Structures and sequences of shRNA and pre-miRNA. Green and red arrowheads indicate DC21 and DC22 cleavages, respectively. (**J**) *In vitro* DICER cleavage assays for RNAs shown in (I). SC: single cleavage products. (**K**) The difference between DC22 and DC21 accuracy was calculated for DICER-WT and DICERΔdsRBD based on data from three repeated assays conducted as shown in (J). *p < 0.05.

To identify the RNA elements recognized by dsRBD, we conducted high-throughput dicing assays for both DICER-WT and DICERΔdsRBD using randomized shRNA sequences. We introduced three randomized base pairs (bp) in six groups (designated as group 1 to group 6) in the region from position 14 to 21 nt from the 5′-end of the shRNA (Figure 1A, Supplementary Figure S1D). We cloned and sequenced the original substrates and their cleaved products (F2, F1-2, and F2-3) (Supplementary Figure S1E). Following this, we utilized the 32N-barcode located in the secondary loop of the original substrate and the cleaved products to map them together, which allowed us to determine the DICER cleavage sites. We repeated the high-throughput dicing three times and obtained a barcode pool for each shRNA sequence in both DICER-WT and DICERΔdsRBD samples (Figure 1B), observing all expected shRNA sequences (Figure 1C). We calculated the total double cleavage efficiency for each variant in each repetition of the high-throughput dicing assays and plotted the values for each variant between repeats 1 and 2, as well as between repeats 1 and 3. The high reproducibility of the three repeated experiments, based on the total double cleavage efficiency, ensures reliable data for further analysis (Figure 1D).

In accordance with our previous high-throughput dicing assays (26), DICER displayed three distinct cleavage patterns: double cleavage (DC), and single cleavage on either the 5′-strand (5′-SC) or the 3′-strand (3′-SC) (Figure 1E). The resulting DC cleavage products displayed a typical DICER activity profile, containing a 2 nt overhang at their 3′-end (Supplementary Figure S1F). DC events were predominantly observed at positions 21 (DC21) and 22 (DC22) from the 5′-end (Figure 1E). Moreover, the deletion of dsRBD led to an increase in DICER's SC activity (Supplementary Figure S1G), which is in line with our earlier findings (26).

We compared the cleavage accuracy and efficiency at DC21 and DC22 between DICER-WT and DICERΔdsRBD (Figure 1F–1H). The results showed that the deletion of dsRBD significantly reduced both the accuracy and efficiency of DC21 (Figure 1F). However, the efficiency of DC22 cleavage was only mildly affected, although its accuracy was increased when compared to the WT (Figure 1G). Notably, the deletion of dsRBD resulted in enhanced DC22-DC21 value (Figure 1H). These findings suggest that dsRBD plays a crucial role in selectively stimulating DC21 and, consequently, reducing the DC22/DC21 ratio.

To confirm the role of dsRBD in DICER cleavage, we conducted tests on shRNA and pre-mir-378a (Figure 1I). The results demonstrated that the absence of dsRBD led to the loss of DC21 cleavage, resulting in an increased DC22–DC21 accuracy value (Figure 1J, 1K). Furthermore, DICERΔdsRBD yields a relatively higher quantity of SC than DICER-WT for the two tested substrates (Supplementary Figure S1H).

### mWCU enhances the DICER cleavage via dsRBD

**Figure 2.**
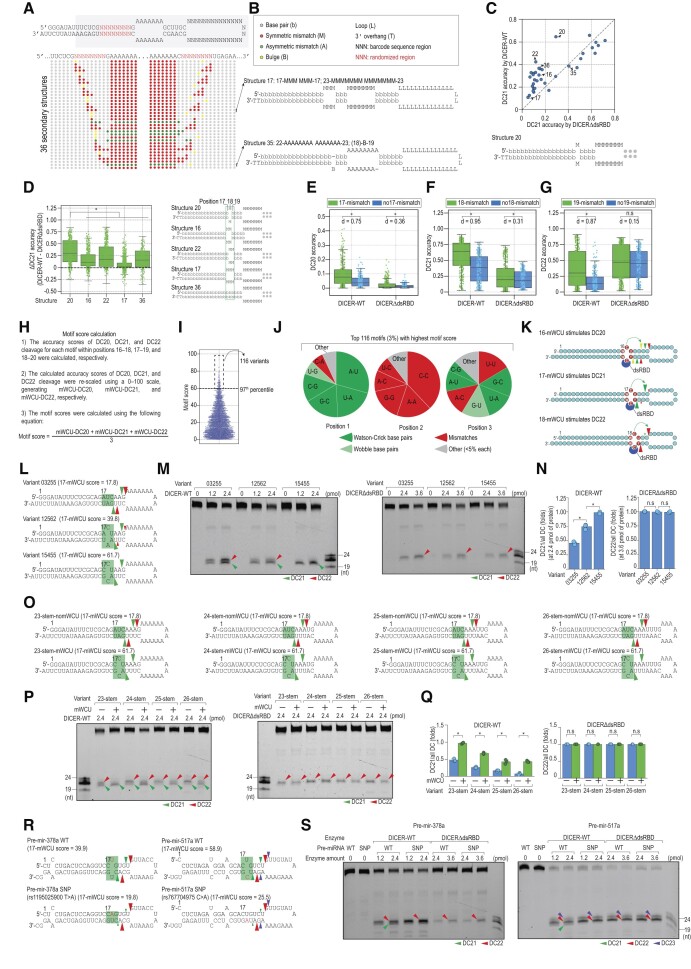
mWCU enhances the DICER cleavage via dsRBD. (**A**) The positional structural profile of 36 shRNA structures. We used RNAfold (47) to predict the secondary structure of 23,296 shRNA variants. The dot-bracket structures obtained from RNAfold were converted into custom-designed structures in which each nt was assigned one of the following letters: L (loop), b (base pair), M (symmetric mismatch), A (asymmetric mismatch), B (bulge) and T (3′-overhang). The gray, red, green, and yellow circles represent b, M, A and B's positional structure, respectively. The randomized regions are shown in red letters. **(B)** Examples of two representative shRNA structures. (**C**) Scatter plot of 36 shRNA structures comparing the DC21 cleavage accuracy between DICER-WT and DICERΔdsRBD. (**D**) Mismatches surrounding the mismatch in position 18 reduce its DC21-enhancing activity. Each dot represents one shRNA variant harboring the indicated structure. (**E–G**) Effect of a single mismatch on DC20, DC21, and DC22 of DICER-WT or DICERΔdsRBD. 17-mismatch, 18-mismatch, and 19-mismatch indicate the structure containing a single mismatch in positions 17, 18 and 19, respectively, with two bp flanking the mismatch. Structures no17-mismatch, no18-mismatch, and no19-mismatch, on the other hand, contain no mismatches. Each dot represents one shRNA variant harboring the indicated structure. *p < 0.05, n.s: not significant. ‘d’ represents Cohen's d value, which quantifies the standardized mean difference between two samples. (**H**) The calculation of averaged accuracy scores. (**I**) The scatter plot showing averaged accuracy scores for motifs identified in the high-throughput dicing assays. Each dot represents one motif. There are 4,096 possible motifs in the 3-bp window; however, only 3,851 motifs derived from shRNA variants that shared a similar 22-bp stem length were used in this analysis. (**J**) The nt composition of the motifs with the highest average accuracy scores. Each position contained 16 possible combinations of two nt on the 5′- and 3′-strands. The first and second nt in the combination, for example, A-U, are on the 5′- and 3′-strands, respectively. The combinations accounting for more than 5% were shown individually, while the rest were combined in the ‘other’ group. Dark green, light green, red, and grey indicate the Watson-Crick bp, wobble bp, mismatches, and other nt combinations (<5%). (**K**) The mWCU regulates DICER cleavage sites in a position-dependent manner. The position of the mWCU motif is determined by the location of the first nt in the 5′–3′ direction of the motif. The yellow, green, and red arrowheads respectively indicate the DC20, DC21, and DC22 cleavages. (**L**) The sequences and diagrams of three shRNAs containing mWCU in 17–19 with varying scores. The green and red arrowheads indicate DC21 and DC22, respectively. (**M, P, S**) *In vitro* DICER cleavage assays. (**N**) Ratios of DC21/all DC or DC22/all DC calculated for DICER-WT and DICERΔdsRBD from three repeated assays conducted in (M). *p < 0.05, n.s: not significant. (**O, R**) shRNA and pre-miRNA sequences and structures. The green and red arrowheads indicate the DC21 and DC22 cleavages, respectively. (**Q**) Ratios of DC21/all DC or DC22/all DC calculated for DICER-WT and DICERΔdsRBD from three repeated assays conducted in (O). *p < 0.05, n.s: not significant.

The results of the high-throughput dicing assays showed that dsRBD selectively stimulates DC21, but not DC22, indicating its preference for interacting with shRNAs at a site essential for DC21. Our objective was to identify additional RNA elements that regulate the function of dsRBD in stimulating DC21. To achieve this, we classified shRNA sequences into different structures, labeled from 1 to 36, using a 6-symbol system (Supplementary Table S13) and assessed the efficiency and accuracy of DICER-WT and DICERΔdsRBD cleavage for each structure (Figure 2A–C, Supplementary Figure S2A).

We compared the DC21 accuracy for DICER-WT and DICERΔdsRBD in each structure. Consistent with the results in Figure 1, DICERΔdsRBD reduced the DC21 cleavage in many identified structures (Figure 2C). Notably, we found that a specific structure (structure 20) containing a single mismatch in position 18 showed the second highest accuracy and highest efficiency for DICER-WT and the greatest reduction in DC21 accuracy and efficiency for DICERΔdsRBD (Figure 2C, Supplementary Figure S2A). This indicates that the single mismatch in position 18 (or 18 mismatch) enhances the DC21 cleavage in the presence of dsRBD in DICER-WT. We also observed that structures with two mismatches in positions 17 and 18 (structure 16) or 18 and 19 (structure 22), or with three mismatches in positions 17, 18 and 19 (structure 17) reduced the stimulatory activity of 18 mismatch on DC21 compared to the single 18 mismatch (structure 20) (Figure 2D). Based on these findings, we propose that the 18 mismatch enhances the ability of dsRBD to stimulate DC21 more effectively. We also found that the mismatches in positions 17 and 19 stimulated DC20 and DC22 of DICER, respectively. However, their stimulatory effects were less potent than the effect observed with the 18 mismatch on DC21 by comparing Cohen's d value, which expresses the standardized mean difference between two samples (Figure 2E–G, Supplementary Figure S2B, C). This is consistent with our observation in Figure 1 that dsRBD preferentially stimulates DC21. Furthermore, we did not observe a notable impact of a single mismatch in any position on DICERΔdsRBD (Figure 2E–G). These findings suggest that the single mismatch selectively affects DICER cleavage sites in the presence of dsRBD.

To gain a deeper understanding of the single mismatch affecting DICER cleavage sites, we first determined the accuracy scores of DICER cleavage at the DC20, DC21, and DC22 for variants containing three base pairs randomized in three regions 16–18, 17–19, 18–20, respectively. These scores were then rescaled to a range of 0–100 by using min-max normalization method, and the averaged accuracy scores of these three regions were calculated (Figure 2H, Supplementary Table S4). The rationale for using averaged accuracy scores, instead of just DC21 accuracy scores, is to provide a more comprehensive understanding of the motif's performance across different positions, reducing the potential bias introduced by analyzing a single position. We focused on the top 3 percentile of the averaged accuracy scores and examined their nt composition (Figure 2I, J). We discovered that these motifs are characterized by a mismatch in the second position, with a high enrichment of C-C. This finding is in line with the above findings showing the strong influence of the single mismatch on the DICER cleavage. The first position primarily contains Watson-Crick bp, with a preference for A-U and U-A over G-C and C-G. In the third position, we saw a blend of Watson-Crick bp, wobble bp, and mismatches, which exhibit a higher presence of U compared to other nt. We did not observe any nt enrichment for the motifs beyond the top 3 percentile (Supplementary Figure S2D). Consequently, we designated these top-ranked motifs as mWCU, where ‘m’ represents the motif containing a mismatch, ‘W’ denotes weak bp (A-U or U-A) in the first position, ‘C’ signifies C-C in the second position, and ‘U’ indicates a mix of combinations with a higher enrichment of U in the third position (Figure 2J).

Our results demonstrated that the presence of mWCU motifs had a significant impact on DICER cleavage accuracy when placed in various positions. Specifically, the cleavage accuracy was predominantly enhanced at corresponding DICER cleavage sites located 4 nt away from the mismatch of the motifs (Figure 2K). In contrast, the influence of mWCU motifs on the cleavage accuracy of DICERΔdsRBD was minimal, regardless of their positions (Supplementary Figures S1E–S2H).

We then evaluated the impact of mWCU on DICER cleavage sites by testing three shRNAs containing mWCU in position 17 with varying scores (Figure 2L). We found that shRNAs with a high-scored mWCU stimulated DC21 more effectively than those with a low-scored mWCU, and this effect was not observed when dsRBD was deleted from DICER (Figure 2M, N). As our previous study indicated, a longer stem favored cleavage at DC22 (26), so we assessed the impact of mWCU on shRNAs with different stem lengths (Figure 2O). We discovered that mWCU could shift the cleavage sites of DICER to DC21 in all tested shRNAs, and again, this effect was not observed with DICERΔdsRBD (Figure 2P, Q, Supplementary Figure S2I). Moreover, we also demonstrated that adding a high-scored mWCU in position 18 stimulated DICER to cleavage at DC22 (Supplementary Figure S2J–L).

To gain a better understanding of mWCU's functions, we examined its impact on the DICER cleavage in human pre-miRNAs. We screened single nucleotide polymorphisms (SNPs) in pre-miRNAs and identified many SNPs that largely altered scores of mWCU (42) (Supplementary Figure S2M, Supplementary Table S9). For validation purposes, we selected two SNPs that lowered the 17-mWCU score in pre-mir-378a from 39.9 to 19.8, and in pre-mir-517a from 58.9 to 25.5 (Figure 2R). We observed that DICER's cleavage of SNP pre-miRNAs, which contained lower 17-mWCU scores, was diminished at DC21 compared to the cleavage of WT pre-miRNAs, which contained higher 17-mWCU scores (Figure 2S). We also utilized the pre-mir-517a backbone to confirm that the C-C mismatch in the 17-mWCU had a more substantial influence on inducing DC21 than the U-C, A-A and G-G mismatches (Supplementary Figure S2N–P). Furthermore, we validated that the A-U in the ‘W’ position of 17-mWCU exerted a stronger impact on inducing DC21 than C-G in the same position (Supplementary Figure S2N–P). It is important to note that the absence of dsRBD significantly reduced DC21 in pre-mir-378a and shRNAs, resulting in a significant increase in the DC22/DC21 ratio. However, unlike the tested shRNAs, in the case of pri-mir-378a, the absence of dsRBD also seemed to reduce DC22 (Figures 1J, 2S), regardless of mWCU motifs. This suggests that dsRBD might also support DC22 cleavage in this pre-miRNA by interacting with different RNA regions other than mWCU.

Together, our findings provide evidence that mWCU plays a critical role in determining the sites of DICER cleavage through dsRBD.

### The YCR determines cleavage sites of DICER independently of dsRBD

**Figure 3.**
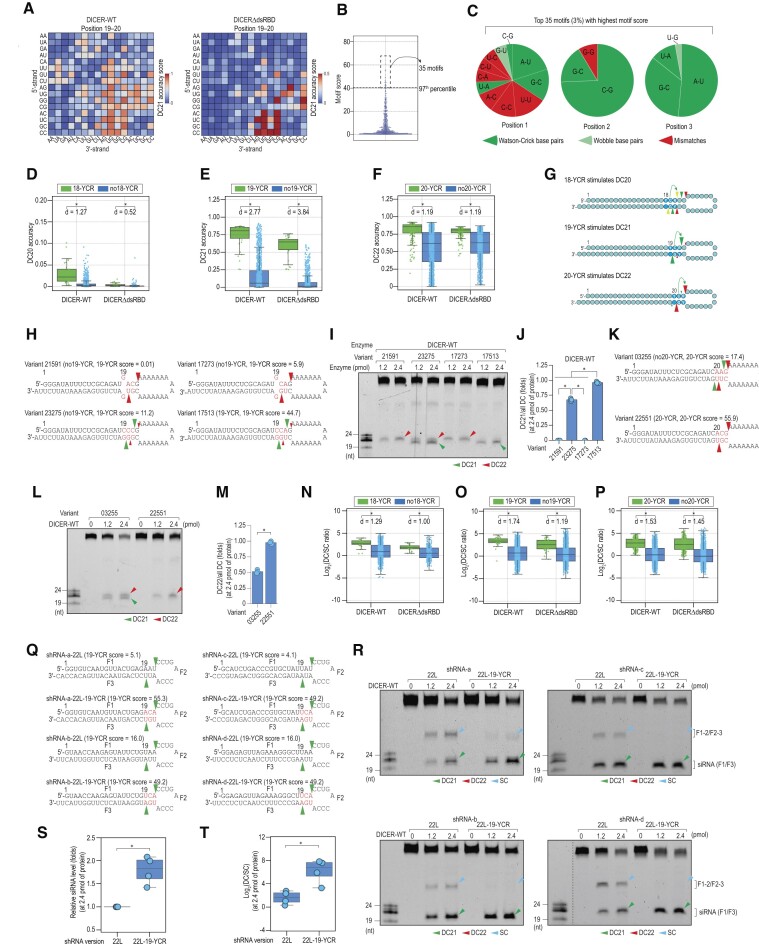
The YCR determines cleavage sites of DICER. (**A**) DC21 accuracy scores of DICER and DICERΔdsRBD for dinucleotide combinations in positions 19–20 of shRNAs. (**B**) The average accuracy scores of the motifs were calculated for DICERΔdsRBD and plotted against motifs in the scatter plot. There are 4,096 possible motifs in the 3-bp window; however, only 1,156 motifs derived from shRNA variants that shared a similar 22-bp stem length were used in this analysis. (**C**) The nt composition of the motifs with the highest average accuracy scores. Each position contained 16 possible combinations of two nt on the 5′- and 3′-strands. The first and second nt in the combination, for example, A-U, are on the 5′- and 3′-strands, respectively. The combinations appearing in the top 35 are shown. Dark green, light green, and red indicate the Watson-Crick bp, wobble bp, mismatches. (**D–F**) The DC cleavage accuracy scores of DICER and DICERΔdsRBD were compared between the YCR and noYCR shRNAs. The numbers in 18-YCR, 19-YCR, and 20-YCR indicate the position of Y in shRNAs. Each dot represents one variant harboring the indicated motif. *p < 0.05. ‘*d*’ represents Cohen's *d* value, which quantifies the standardized mean difference between two samples. (**G**) The YCR controls DICER cleavage sites in a position-dependent manner. The position of the YCR motif is determined by the location of the first nt in the 5′–3′ direction of the motif. The yellow, green, and red arrowheads respectively indicate the DC20, DC21 and DC22 cleavages. (**H, K, Q**) Structures and sequences of shRNAs. The green and red arrowheads indicate the DC21 and DC22 cleavages, respectively. (**I, L, R**) *In vitro* DICER cleavage assays. SC: single cleavage products. (**J, M**) Ratios of DC21/all DC (J) or DC22/all DC (M) cleaved products calculated for DICER-WT and DICERΔdsRBD from three repeated assays conducted in (**I**) and (**L**). *: *p* < 0.05. (N–P) Ratios of DC/SC cleaved products calculated for DICER-WT and DICERΔdsRBD from three repeated high-throughput dicing assays. Each dot represents one variant harboring the indicated motif. *: *p* < 0.05. ‘d’ represents Cohen's d value, which quantifies the standardized mean difference between two samples. (**S, T**) Relative siRNA level and DC/SC of the cleaved products were calculated for DICER-WT from cleavage assays conducted in (R). One dot represents one shRNA variant. *p < 0.05.

To explore potential sequence motifs that could influence DICER cleavage accuracy, we analyzed the cleavage accuracy at DC21 of shRNAs containing two consecutive nt on both the 5′- and 3′-strands within the 14–21 region (Figure 3A and Supplementary Figure S3A). Our analysis uncovered that the presence of certain dinucleotide combinations in positions 19–20 significantly enhanced cleavage accuracy at DC21 for both DICER-WT and DICERΔdsRBD (Figure 3A). Conversely, the combinations of dinucleotides in other positions did not exhibit much impact on DICER cleavage sites (Supplementary Figure S3A). This finding implies that there may be sequence motifs containing positions 19–20 that affect DICER cleavage accuracy independently of dsRBD.

To identify potential sequence motifs that influence DICER cleavage sites independently of dsRBD, we focused on analyzing the data obtained from DICERΔdsRBD. We calculated the accuracy scores of each motif for DICERΔdsRBD at different cleavage sites, DC20, DC21 and DC22 for variants randomized in the regions of 18–20, 19–21, 20–22, respectively. We only selected the motifs residing in shRNA variants harboring 22-bp stem structures, therefore, many trinucleotide combinations were excluded from the analysis. Subsequently, we rescaled the accuracy scores at each cleavage site to a scale of 0–100 and calculated the average accuracy scores for each motif across the three cleavage sites (Figure 3B). By selecting the top 3% of motifs with the highest average scores, we identified their nt composition (Figure 3B, C). The first position contains various combinations of nt between the 5′ and 3′-strands, excluding R-R combinations (R is A or G). The second position is highly enriched with C-G pairs, while the third position is enriched with G-C or A-U pairs. Such enrichments were not observed for the remaining 1,121 motif (Supplementary Figure S3B). We named these top motifs as YCR, where Y represents a combination containing at least one Y (Y is C or U), C represents a C-G pair, and R represents R-Y pairs (G-C and A-U). We also considered these calculated scores as YCR scores (Supplementary Table S5).

To evaluate whether these YCR motifs control DICER cleavage accuracy, we categorized shRNAs into two groups based on the presence of YCR motifs: noYCR and YCR in different positions, 18–20. We found that the YCR motifs placed in position 18, 19, and 20 enhanced the cleavage accuracy of DC20, DC21 and DC22 of DICER and DICERΔdsRBD, respectively (Figure 3D–3F, Supplementary Figure S3C). The impact of YCR on DC20 was detected despite the lower DC20 level of DICER. Collectively, these findings led us to propose a function for YCR motifs in controlling DICER cleavage sites, as illustrated in Figure 3G.

To further evaluate the impact of YCR motifs on DICER cleavage sites based on the calculated YCR scores, we introduced motifs with varying YCR scores into an shRNA construct in the position 19 (Figure 3H) and assessed their impact on DICER-WT and DICERΔdsRBD. We found that the DC21 accuracy scores increased with the YCR scores for both DICER-WT and DICERΔdsRBD (Figure 3I, J, Supplementary Figure S3D, E). We also observed that YCR in position 20 (20-YCR) stimulated cleavage at DC22 by DICER-WT (Figure 3K–M). To rule out the possibility that the motif identified in the high-throughput dicing assays using the two-loop shRNA system might function differently between two-loop and one-loop shRNAs, we conducted a side-by-side comparison of the effects of 19-YCR in both types of shRNAs. We observed that the 19-YCR similarly controlled the cleavage sites of DICER in both shRNAs (Supplementary Figure S3F–H). It is important to note that the efficiency of DICER in the two-loop shRNAs appeared to be lower than that in one-loop shRNAs (Supplementary Figure S3F–H). This finding is consistent with previous studies (23,34) showing that loop size matters for DICER cleavage efficiency. This evidence further supports our conclusion that the secondary loop may not affect the impact of identified motifs in the stem or loop (from our previous study) on determining DICER cleavage sites.

### The YCR reduces single cleavage of DICER independently of dsRBD

We investigated the effect of the YCR motif on the single cleavage of DICER. Our results showed that the YCR motifs in positions 18, 19 and 20 increased the DC/SC ratios of both DICER and DICERΔdsRBD (Figure 3N–P). Our findings suggest that YCR controls DICER single cleavage in a dsRBD-independent manner.

In order to further investigate the effect of YCR on shRNA cleavage by DICER, we incorporated 19-YCR into several shRNA backbones with a loop positioned at 22 (Figure 3Q), which is known to facilitate DC21 cleavage (26). This allowed for coordination between the loop and 19-YCR, resulting in increased DC21 cleavage and reduced single cleavage for both DICER-WT and DICERΔdsRBD, consistent with the high-throughput dicing assays (Figure 3R–T, Supplementary Figure S3I–K). Additionally, our results showed that the incorporation of 19-YCR into shRNAs increased their knockdown efficiency in reporter assays (Supplementary Figure S3L, M).

### The conserved YCR motifs determine cleavage sites of DICER in pre-miRNAs

**Figure 4.**
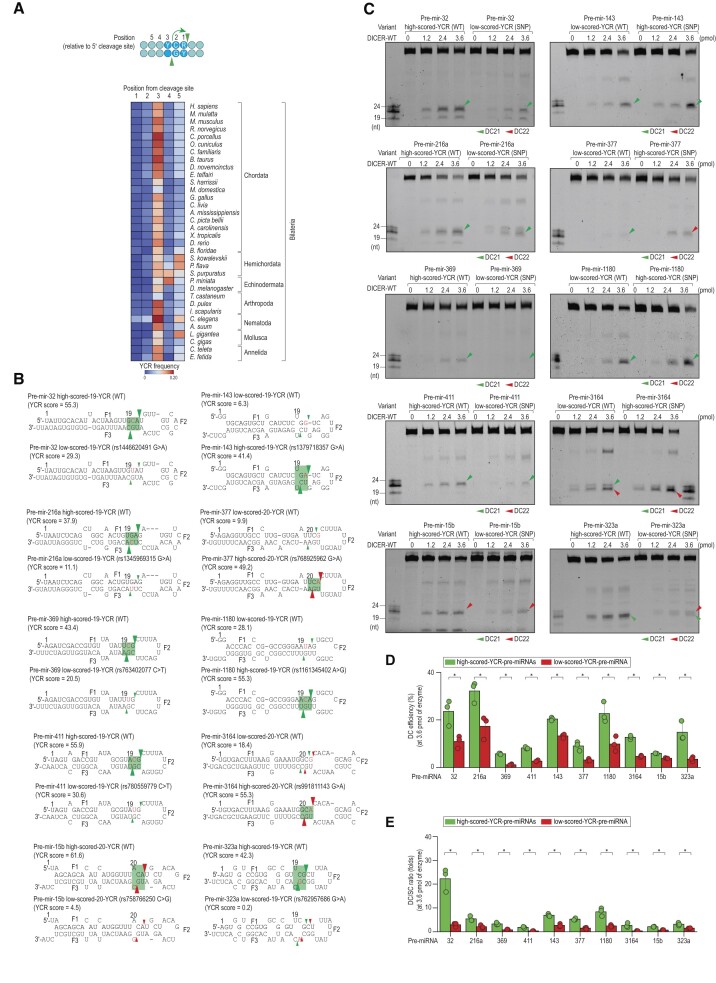
Validate functions of YCR in determining cleavage sites of DICER. (**A**) Enrichment of YCR in pre-miRNAs of humans and other animals. The YCR frequency was calculated as the ratio of pre-miRNAs containing this motif to all pre-miRNAs. Blue circles indicate YCR motifs. (**B**) Structures and sequences of pre-miRNAs. The green and red arrowheads indicate the DC21 and DC22 cleavages, respectively. The SNP nt are indicated in red. (**C**) *In vitro* DICER cleavage assays. The green and red arrowheads indicate the DC21 and DC22 cleavages, respectively. (D, E) The DC cleavage efficiency (**D**) and ratios of DC/SC (**E**) of cleaved products calculated for DICER-WT from three repeated assays conducted in (C). The DC cleavage efficiency was measured as a ratio of the DC product to the pre-miRNA. *P < 0.05.

Based on our findings, it appears that the YCR motif is located 3 nt upstream of the DICER cleavage site on the 5′-strand and stimulates its corresponding cleavage site. Therefore, we investigated the prevalence of YCR in human pre-miRNAs located 1–5 nt upstream of the DICER cleavage sites. Our results show that YCR motifs are enriched in human pre-miRNAs at the 3 nt upstream of DICER cleavage sites (Figure 4A, Supplementary Table S11). Interestingly, these motifs are also conserved in pre-miRNAs from various animal species in similar positions (Figure 4A). These findings suggest that YCR may be a conserved feature in animal pre-miRNAs. We also conducted an analysis for mWCU in human and other animal pre-miRNAs and found that it was not enriched in these animals (Supplementary Figure S4A, Supplementary Table S12).

To investigate the function of YCR, we analyzed SNPs in human pre-miRNAs and identified several SNPs that either significantly increased or decreased the scores of YCR motifs (Supplementary Figure S4B, Supplementary Table S10) (42). We selected 10 of these SNPs (Figure 4B) for further examination. Our findings revealed that the SNP-created high-scored YCR motifs promoted DC and reduced SC. In contrast, the SNP-created low-scored YCR motifs led to a decrease in DC but an increase in SC by DICER (Figure 4C–E). The comparable effects of YCR were also evident with these pre-miRNAs for DICERΔdsRBD (Supplementary Figure S4C–E). These findings suggest that YCR has a vital function in modulating the cleavage activity of DICER in human pre-miRNAs, regardless of dsRBD.

To exclude the chance that the discovered motif in the high-throughput dicing assays utilizing the two-loop shRNA system might have varied functionality between two-loop and one-loop pre-miRNAs, we performed a simultaneous evaluation of the consequences of 19-YCR in both categories of pre-miRNAs. We noticed that the 19-YCR comparably boosted the cleavage efficacy of DICER in both pre-miRNAs (Supplementary Figure S4F–H). This additional proof reinforces our assertion that the secondary loop may not influence the effect of detected motifs in the stem.

### Molecular basis of motif recognition

In order to further confirm that mWCU is dependent on dsRBD while YCR is not, and to elucidate the molecular basis for mWCU recognition, we closely examined the existing DICER/RNA structural model (21), with a particular focus on identifying which residues interact with mWCU. Our structural analysis discovered that multiple residues in dsRBD, specifically S1852, R1855, E1859, R1898 and K1901, could potentially establish direct contact with RNAs via hydrogen bonds (Supplementary Figure S5A). Notably, our analysis showed that R1855 forms three hydrogen bonds with the C-C mismatch (19C-53C in the structure model), a C mismatch component of the 18-mWCU motif. We observed only one hydrogen bond between R1855 and 20G, a common component of both 18-mWCU and 20-YCR motifs. Moreover, E1859 forms a single hydrogen bond with 55U, which lies beyond the scope of both our defined motifs. We also noted that each of the S1852 and K1901 residues in dsRBD interact with the phosphodiester backbone in the 20G position (shared by the two motifs) via a single hydrogen bond, and R1898 interacts with the diester linkage at 28G, which does not belong to either mWCU or YCR. Additionally, R1855 appears to be conserved across different animal species (Supplementary Figure S5B). A comparison of the dsRBDs of human DICER with the resolved dsRBD structure of mice, and the predicted structures of flies and worms, shows that R1855 occupies a similar position in these dsRBDs (Supplementary Figure S5C). This conservation information, coupled with the observation that R1855 was the only residue seen to interact with the bases (C-C mismatch) in mWCU, suggests that its interaction with the C-C mismatch in 18-mWCU might significantly contribute to the dsRBD-18mWCU interaction.

**Figure 5.**
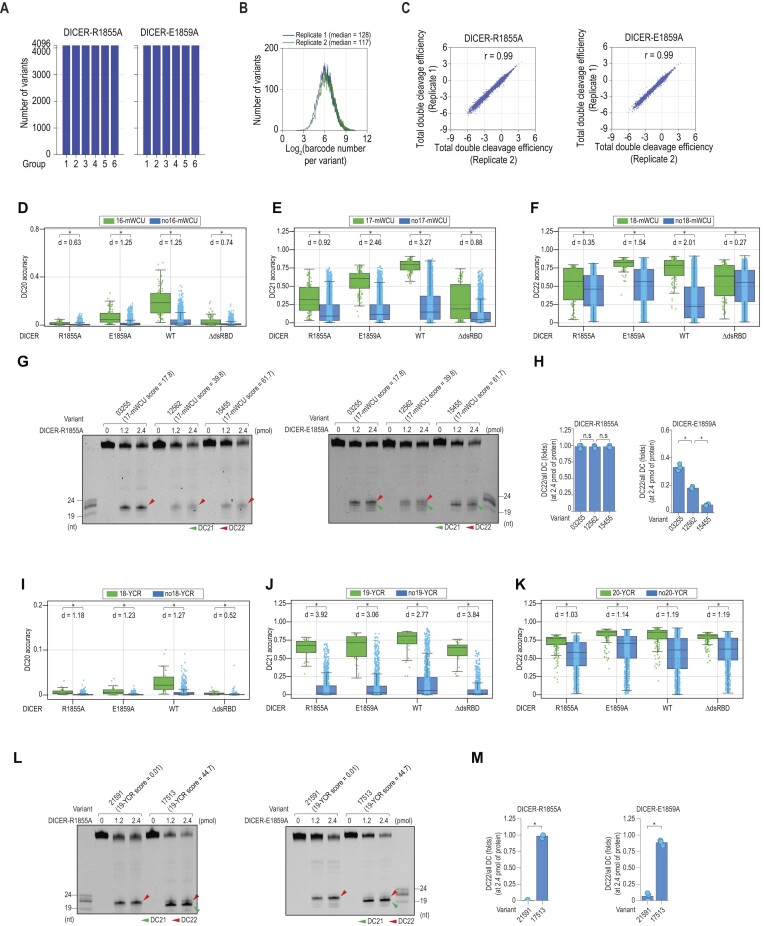
Molecular basis of motif recognition. (**A**) Identification of synthesized shRNA variants in the high-throughput dicing assays for DICER-R1855A and DICER-E1859A. Blue bars indicate the number of identified shRNA variants in each group. 4,096 represents all possible variants in each group. (**B**) Distribution of log_2_(barcode numbers) for shRNA variants identified in two repeated high-throughput dicing assays. (**C**) Reproducibility of the two repeated high-throughput dicing assays. The double cleavage efficiency was estimated for each variant in each repetition of the assays. Each dot in the plots represents one shRNA variant. R is Pearson's correlation coefficient. (**D–F**) Effect of the mWCU motif on DC20, DC21 and DC22 of different DICER variants. The numbers in 16-mWCU, 17-mWCU, and 18-mWCU indicate the position of W in shRNAs. Each dot represents one shRNA variant harboring the indicated structure. *p < 0.05, n.s: not significant. ‘*d*’ represents Cohen's *d* value, which quantifies the standardized mean difference between two samples. (**G, L**) *In vitro* cleavage assays for DICER-R1855A and DICER-E1859A. The green and red arrowheads indicate the DC21 and DC22 cleavages, respectively. (**H, M**) Ratios of DC22/all DC for cleaved products calculated for DICER-R1855A and DICER-E1859A from three repeated assays conducted in (G) and (L). *p < 0.05, n.s: not significant. (**I–K**) The DC cleavage accuracy scores of DICER variants were compared between the YCR and noYCR shRNAs. The numbers in 18-YCR, 19-YCR, and 20-YCR indicate the position of Y in shRNAs. Each dot represents one variant harboring the indicated motif. *p < 0.05. ‘*d*’ represents Cohen's *d* value, which quantifies the standardized mean difference between two samples.

Considering a previous study suggested that two residues, R1855 and E1859, interact with the mismatch in its identified ‘GYM’ motif (21,27), which corresponds to the mismatch in our mWCU, we generated two DICER mutants, R1855A and E1859A, by replacing R or E with A (Supplementary Figure S5D). We then conducted high-throughput dicing assays for these two mutant proteins using the shRNA library shown in Figure 1A. Analysis of the high-throughput dicing assays identified all expected shRNA variants (Figure 5A). Additionally, the results of two assay replicates were consistent, as indicated by the barcode number per variant in the two replicates (Figure 5B) and the consistency in cleavage efficiency calculation (Figure 5C).

We examined the impact of mWCU on these two mutant proteins and found that the influence on E1859A was similar to the DICER-WT, while on R1855A, it was similar to DICERΔdsRBD (Figure 5D–F). The impact of mWCU in different positions on controlling DICER cleavage sites was significantly reduced in R1855A, and mildly reduced in E1859A (Figure 5D–F). This supports our hypothesis that R1855 is crucial for recognizing mWCU, while E1859 contributes minimally to mWCU recognition. Further, we validated our high-throughput dicing assay results by conducting cleavage assays for these two mutant proteins with different shRNA variants featuring various mWCUs in different positions (Figure 5G, H). We found that mWCU did not cause a change in cleavage sites of R1855A, similar to DICERΔdsRBD. Yet, it still caused cleavage site alterations in E1859A, like DICER-WT. This further demonstrated that R1855 recognizes mWCU.

Subsequently, we evaluated the influence of YCR on two DICER mutant proteins and discovered that YCR could shift the cleavage sites of these mutant proteins, in a manner comparable to DICER-WT or DICERΔdsRBD as seen in both the high-throughput (Figure 5I–K) and validated cleavage assays (Figure 5L, M). These biochemical findings bolster our assertion that YCR regulates the DICER cleavage sites, independently of dsRBD.

We further investigated the influence of R1855A, E1859A and DICERΔdsRBD by conducting rescue experiments on HCT116 DICER-KO cells. Upon analyzing the small RNA sequencing results, we found that R1855A, E1859A and DICERΔdsRBD did not affect YCR miRNA expression in comparison to DICER-WT (Supplementary Figure S5E). We also analyzed the rescue experiment conducted on HEK293T DICER-KO cells from the previous study (27) and found that DICERΔdsRBD did not cause changes in YCR miRNA expression compared with DICER-WT (Supplementary Figure S5F). These findings further substantiate that dsRBD is not essential for YCR's role in altering DICER cleavage sites. We did not compare the expression of mWCU miRNAs with no-mWCU miRNAs in our rescue experiments, as only a single mWCU miRNA was detected in our rescue experiments (Supplementary Figure S5G). However, in the published rescue experiments (27), we detected three mWCU miRNAs and found that miRNAs containing 17-mWCU showed more significant reductions in DC21 isomiR compared to those without 17-mWCU in DICERΔdsRBD (Supplementary Figure S5H).

Our prior research suggests that dsRBD is crucial for double cleavage or for positioning RIIIDa and RIIIDb in shRNAs (26). In this study, we evaluated the single cleavage activity of different DICER variants. We observed that DICERΔdsRBD increased 3p-single cleavage, implying that dsRBD might play a significant role in positioning the 5p-cleavage domain, RIIIDb (Supplementary Figure S5I). Additionally, we examined the single cleavage of R1855A and E1859A and discovered that neither of them altered the single cleavage level when compared with the DICER-WT, suggesting that these two residues primarily serve to anchor DICER to shRNAs via the mWCU motif (Supplementary Figure S5J). Other parts of dsRBD might be crucial for positioning DICER.

### Two-motif model for explaining DICER's cleavage site selection

**Figure 6.**
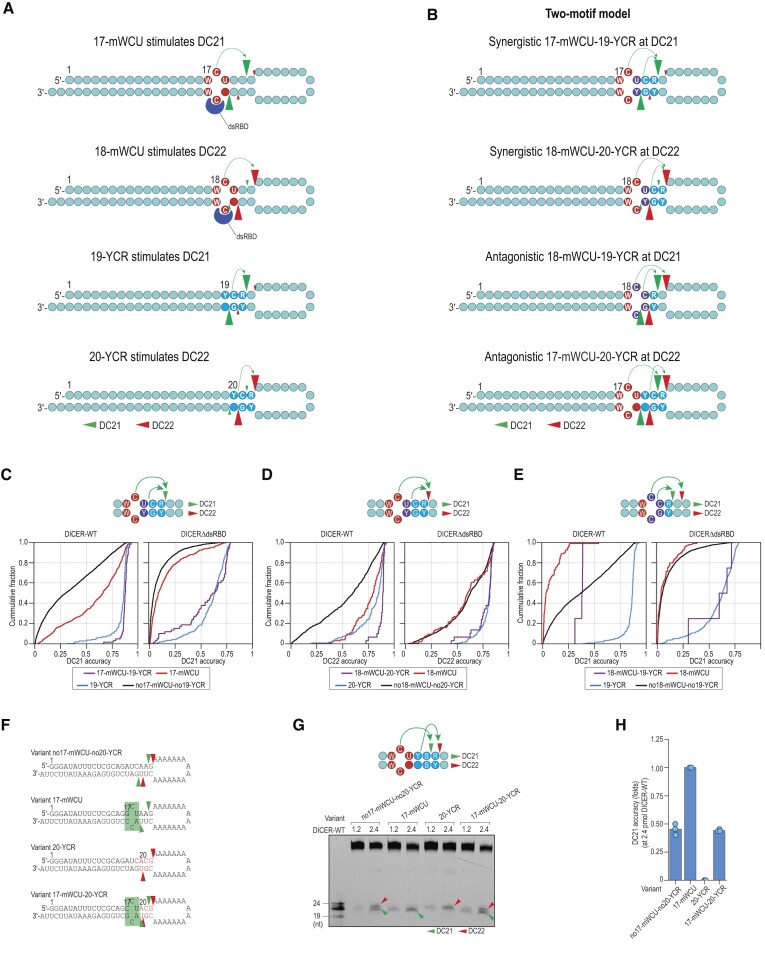
Two-motif model for explaining DICER's cleavage site selection. (**A**) mWCU and YCR control DICER cleavage sites in a position-dependent manner. The position of each motif is determined by the location of the first nt in the 5′–3′ direction of each motif. Red circles represent the mWCU motif, while blue circles symbolize the YCR motif. Purple circles indicate shared positions between the two motifs. Green arrowheads represent the DC21 cleavage, and red arrowheads denote the DC22 cleavage. (**B**) Two-motif model explains DICER's cleavage sites. Model 1: 17-mWCU and 19-YCR are synergistic in stimulating DC21. Model 2: 18-mWCU and 20-YCR are synergistic in stimulating DC22. Model 3: 18-mWCU and 19-YCR are antagonistic, with 18-mWCU stimulating DC22 while 19-YCR stimulates DC21. Model 4: 17-mWCU and 20-YCR are antagonistic, with 17-mWCU stimulating DC21 while 20-YCR stimulates DC22. (**C**) Cumulative plots showing 17-mWCU and 19-YCR are coordinated in stimulating DC21. (**D**) Cumulative plots showing 18-mWCU and 20-YCR are coordinated in stimulating DC22. (**E**) Cumulative plots showing 18-mWCU and 19-YCR are not coordinated in stimulating DC21 and DC22. (**F**) Structures and sequences of shRNAs. The green and red arrowheads indicate the DC21 and DC22 cleavages, respectively. (**G**) *In vitro* DICER cleavage assays. (**H**) Ratios of DC21/all DC calculated for DICER-WT from three repeated assays conducted in (F). *p< 0.05.

Our two-motif model suggests that mWCU and YCR are distinct motifs that influence DICER cleavage sites at DC21 and DC22 depending on their positions in shRNAs/pre-miRNAs. mWCU is dependent on dsRBD, but YCR is not (Figure 6A). We hypothesized that these two motifs may exhibit cooperative or non-cooperative roles in stimulating DC21/DC22 cleavage, which could vary depending on their respective locations (Figure 6B).

To validate our two-motif model, we analyzed the high-throughput dicing assays and identified three out of the four arrangements of the two motifs. Our results showed that 17-mWCU and 19-YCR acted synergistically to enhance the DC21 cleavage of DICER-WT, outperforming 17-mWCU or 19-YCR alone. 19-YCR increased DC21 cleavage of DICERΔdsRBD, but 17-mWCU did not stimulate DC21 cleavage of DICERΔdsRBD, nor did it cooperate with 19-YCR to stimulate DC21 cleavage of DICERΔdsRBD. These findings support a model of synergistic mWCU-YCR at DC21 and suggest that mWCU but not YCR is dependent on dsRBD (Figure 6C).

Similarly, 18-mWCU and 20-YCR cooperated to stimulate the DC22 cleavage of DICER-WT, surpassing 18-mWCU or 20-YCR alone. While 20-YCR still stimulated the DC22 cleavage of DICERΔdsRBD, 18-mWCU neither stimulated the DC22 cleavage of DICERΔdsRBD nor cooperated with 20-YCR to stimulate the DC22 cleavage of DICERΔdsRBD. These results support a model of synergistic mWCU-YCR interaction at DC22 and suggest that mWCU, but not YCR, is dependent on dsRBD (Figure 6D).

Furthermore, we found that 18-mWCU reduced the DC21 cleavage of DICER-WT and counteracted the stimulatory effect of 19-YCR on the DC21 cleavage of DICER-WT. Moreover, 18-mWCU did not affect the DC21 cleavage of DICERΔdsRBD and did not counteract the stimulatory effect of 19-YCR on the DC21 cleavage of DICERΔdsRBD. These findings support a model of antagonistic mWCU-YCR, and suggest that mWCU but not YCR is dependent on dsRBD (Figure 6E).

Since our high-throughput dicing assays did not contain the fourth arrangement of the two motifs spanning a 6-bp window, we synthesized four shRNAs containing this arrangement (Figure 6F). We found that 20-YCR stimulated DC22 cleavage, while 17-mWCU induced DICER to cleave at DC21. The presence of both motifs caused DICER-WT to cleave the shRNA at both DC21 and DC22 (Figure 6F, G). These findings support a model of antagonistic mWCU-YCR.

In summary, our findings demonstrate that the specific arrangement of the two motifs plays a critical role in DICER's cleavage activity, and our experiments using shRNAs further support the validity of our models.

## Discussion

In this study, we utilized a two-loop shRNA system to investigate the activity of DICER, simultaneously examining the cleavage sites and the efficiency of DICER by sequencing both substrates and cleaved products. The barcodes in the secondary loop allowed us to map the cleaved products to the substrate, enabling us to examine the randomized region at and surrounding DICER's cleavage sites. Some may express concerns about using the nonclassical two-loop shRNA system, as it could potentially influence DICER cleavage differently from the classic one-loop structure in the high-throughput dicing assays. This is attributed to DICER's preference for medium-sized loops that fit into an internal pocket (33,34). However, it is important to note that while DICER has an optimal substrate when the loop fits into the internal pocket, it can still cleave pre-siRNAs that do not contain the loop (44). Therefore, the loop could be considered dispensable for the DICER cleavage. Additionally, our two-loop shRNA design positions the randomized sites in the stem, ensuring that all randomized two-loop shRNAs share a similar two-loop structure. Consequently, the effect of the two loops on the DICER cleavage should be consistent across all randomized shRNAs. This uniformity allows us to eliminate the influence of the two loops when comparing the impact of different randomized regions in the stem on DICER cleavage. Furthermore, we have successfully demonstrated in our previous report (26) and this study that numerous findings using the two-loop shRNA system have been validated with one-loop shRNAs and pre-miRNAs.

Additionally, conducting high-throughput dicing assays for DICER-WT and dsRBD mutants in parallel allowed us to investigate the roles of dsRBD in determining DICER cleavage efficiency and accuracy for each shRNA sequence. Based on our high-throughput dicing assays and validated experiments, we propose a two-motif working model in which mWCU and YCR are distinct motifs that influence DICER's cleavage sites in different ways. It's crucial to understand that the motifs should be interpreted as a combination of three consecutive pairs, all of which are detailed in Supplementary Tables S4 and S5. Their ability to promote the DICER cleavage is scored based on the high-throughput dicing data. Our study provides compelling evidence to support this model. Firstly, mWCU is dependent on dsRBD, while YCR is not. Secondly, these two motifs can coordinate to stimulate a single DICER cleavage site, but if not coordinated, they prompt DICER to cleave at different positions. Finally, our model explains the miRNA expression of DICER-KO cells rescued by DICER and DICERΔdsRBD, showing that only mWCU miRNAs, not YCR miRNAs, exhibit a significant reduction in expression upon dsRBD deletion. In our recent study, we identified sequence motifs, known as DRES, that are specifically recognized by DROSHA, another RNase III enzyme in humans (45). The fact that both DROSHA and DICER, two RNase III enzymes, recognize specific sequence motifs raises several intriguing aspects for future studies. For example, it is possible that they employ a common mechanism for motif recognition, or that RNase III enzymes from diverse species may also possess the ability to recognize similar sequence motifs.

The functionality of mWCU depends on the dsRBD of DICER, as it facilitates DICER cleavage, and is analogous to that of mGHG in DROSHA. Both motifs are recognized by the dsRBD of their respective proteins, and this recognition has been supported by biochemical and structural studies (21,27,46–49). The mGHG and mWCU motifs both feature a mismatch in the middle position. While mGHG is depleted of G–G mismatches, mWCU still accepts G–G mismatches and is enriched with C–C mismatches. These two motifs differ mainly in their flanking positions around the mismatch. Specifically, in the first position, mWCU is enriched with A–U or U–A pairs, while mGHG is enriched with C–G and U–G pairs. In the third position, mWCU can contain mismatches and bp. In contrast, mGHG predominantly features bp, mostly a C–G pair in the third position. These similarities, in addition to structural similarities (50), support the hypothesis that DICER and DROSHA evolved from a common origin. However, the variations in these two motifs also imply that these proteins have evolved to serve distinct functions (1,2). The fact that mWCU significantly influences DICER cleavage sites and its recognition mechanism might be conserved across many animals, yet, it is not enriched in many animal pre-miRNAs. One explanation for this is that the region containing mWCU may also be utilized by AGO. For instance, the presence of an mWCU with a mismatch in could destabilize the 3p-end of miRNA duplex, facilitating 3p miRNA selection by AGO. Secondly, mWCU bears resemblance to the mGHG motif. Its presence near DICER cleavage sites might also have some affinity to DROSHA, causing DROSHA to cleave pri-miRNAs at unproductive sites. While the mWCU motif is not as highly enriched in pre-miRNAs as the mGHG motif is in pri-miRNAs, it still plays a vital role in miRNA biogenesis for a subgroup of miRNAs containing it. Additionally, our findings suggest that the mWCU motif may play a more critical role in regulating miRNA functions. We demonstrated that SNPs occurring in this motif can alter DICER's cleavage sites, potentially leading to different miRNA isoforms.

This research, along with the study (26), suggests a dual role for dsRBD in both tethering and positioning DICER on RNA substrates. Our study provides evidence that mWCU influences DICER cleavage sites via dsRBD, specifically through the interaction between R1855 and a mismatch in mWCU. This finding underscores the function of dsRBD in tethering DICER to precise positions in shRNAs/pre-miRNAs via mWCU motifs. Previous research has shown that dsRBD also aids in positioning the RIIIDa and RIIIDb domains of DICER in RNAs, facilitating double cleavage. In this study, we further revealed that dsRBD seems to have a more significant role in positioning RIIIDb in RNAs than RIIIDa. Interestingly, we discovered that R1855, while essential for dsRBD's tethering function, is not required for its positioning function. Future investigations into other parts of dsRBD may pinpoint which residues are critical for its tethering functionality. While mWCU depends on dsRBD, YCR operates independently of dsRBD. Surrounding this motif, there are three domains with RNA-binding affinity: RIIIDa, RIIIDb, and helicases. Future research involving a structure of full-length DICER/RNA (noting that the most recently reported structure does not include helicases) could provide further insights. Additionally, our analysis indicated that most animals use pre-miRNAs enriched in YCR, but not mWCU, to regulate DICER cleavage. This suggests that YCR is the primary motif utilized by these animals. Notably, some animals do not show a significant enrichment of YCR. Further research is needed to understand why different animal pre-miRNAs employ diverse strategies to regulate DICER cleavage.

Recently, a study reported high-throughput dicing assays of DICER using the pre-let-7a-1 substrate model to investigate motifs that control DICER cleavage efficiency (27). The study found that the GYM motif, located 4 nt away from the cleavage site, enhances DICER cleavage efficiency. In the GYM motif, ‘G’ represents a C–G pair, ‘Y’ represents a paired pyrimidine nt on the 3′-strand, and ‘M’ indicates a mismatch. As discussed earlier, our two-motif model spans 4–6 positions, while the GYM model contains only 3 positions. We discovered that the GYM motif can be considered a component of the combined mWCU and YCR motifs when they operate in a coordinated mode (Supplementary Figure S6A). Specifically, when mWCU and YCR are coordinated to form a 5-position motif, its component in positions 2–4 (counting 5′–3′ on the 3′-strand) is similar to the GYM motif (Supplementary Figure S6A). When mWCU and YCR motifs are not coordinated, they form a 6-position motif; in this case, GY and M are separated by one position (Supplementary Figure S6B). In the 4-position uncoordinated model, M and Y merge (Supplementary Figure S6C). Therefore, in the uncoordinated cases, the GYM motif is not found in our model. A possible explanation for the findings in our two-motif model and the GYM model (27) is that our study examined the impact of randomized nt not only on the cleavage efficiency but also on the cleavage sites of DICER. This comprehensive approach allowed us to uncover the cooperative and uncooperative relationships between mWCU and YCR motifs, providing a more detailed understanding of the sequence determinants governing DICER cleavage, meanwhile, the previous study focused only on the cleavage efficiency of DICER in high-throughput dicing assays.

DICER is responsible for recognizing the 5′- and 3′-ends of RNA substrates to determine their length; however, this measurement is imprecise and cannot accurately determine whether 21 or 22 nt should be cleaved from the ends (12–21). As a result, DICER requires multiple RNA elements to fine-tune its cleavage sites. Previous studies have identified several secondary RNA elements that control DICER cleavage, including stem length, loop, and bulge (26). In this study, we have identified two additional RNA motifs, mWCU and YCR, that coordinate or independently determine the cleavage sites of DICER. RNA modifications, editing, or SNPs (as shown in this study) may affect these RNA elements, thereby controlling DICER cleavage sites. Understanding how these RNA-modifying mechanisms control miRNA biogenesis will be crucial for understanding cellular processes and human diseases and should be the focus of future studies.

## Supplementary Material

gkad1186_supplemental_files

## Data Availability

The sequencing data and processed data are deposited at Gene Expression Omnibus (GEO), accession number: GSE247119. Original code for data analysis is deposited at Zenodo (DOI:10.5281/zenodo.8394675).
