# Supplementary material for: Two-motif model illuminates DICER cleavage preferences
Source: Nucleic Acids Res. 2024 Jan 3;52(4):1860–77. doi: 10.1093/nar/gkad1186 (PMC10899750; doi:10.1093/nar/gkad1186)
Supplement: gkad1186_supplemental_files [file gkad1186_supplemental_files.zip › Dicer-two-motif-Supplementary-Figure-S1-S6-v2-only-SupFigs.pdf]

SUPPLEMENTARY DATA

Two-motif model illuminates DICER cleavage preferences

Cong Truc Le, Trung Duc Nguyen, and Tuan Anh Nguyen

Supplementary Figure S1

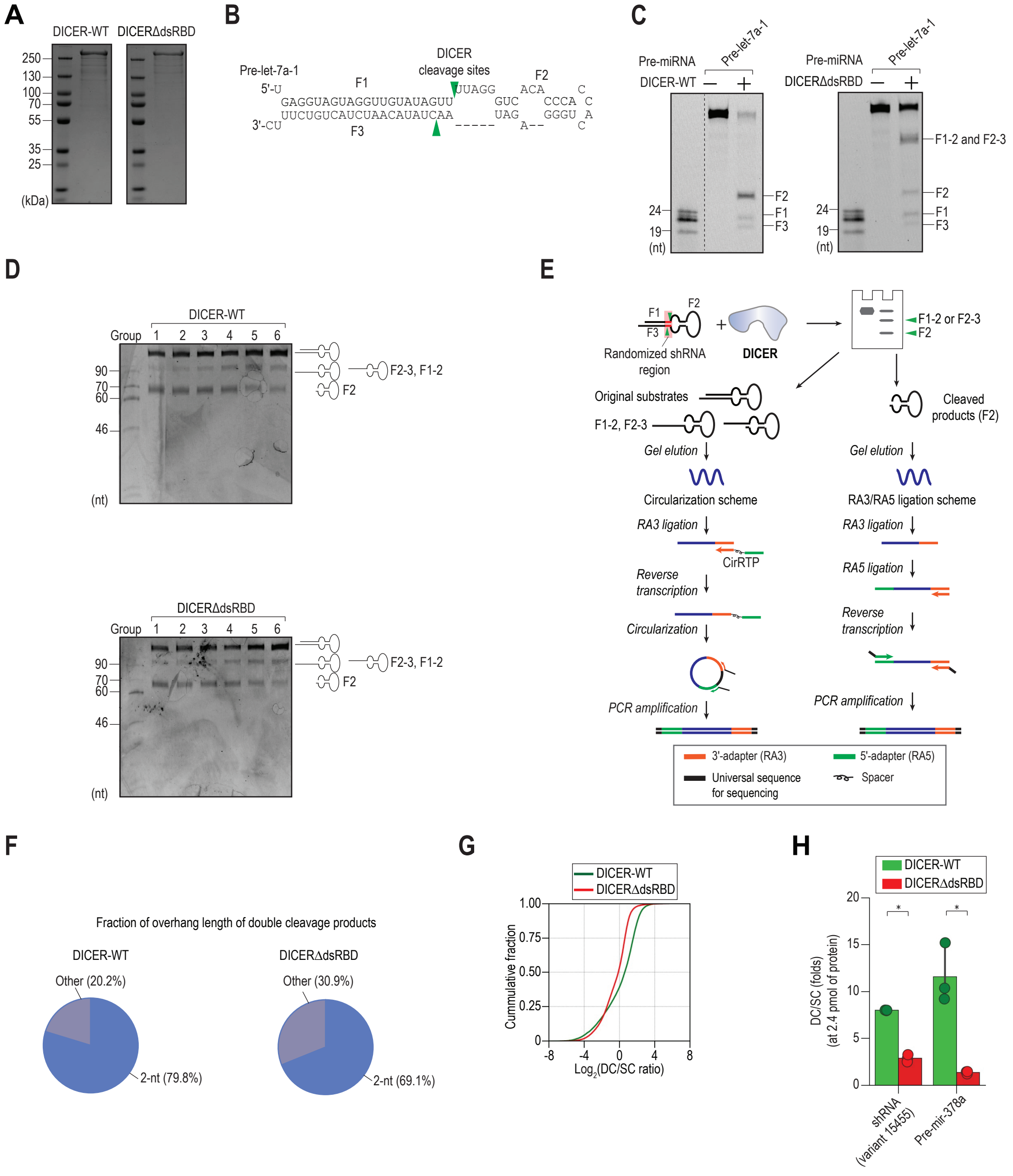

Supplementary Figure S1. dsRBD facilitates DICER DC21 cleavage.
(**A**) The purity of DICER-WT and DICERΔdsRBD was assessed by SDS-PAGE. (**B**) The structure and sequence of pre-let-7a-1, with cleavage sites of DICER indicated by green arrowheads. (**C**) *In vitro* cleavage assays for pre-let-7a-1 were performed using DICER-WT and DICERΔdsRBD. F1-2 and F2-3 are the cleaved products resulting from the single cleavage on the 3'-strand and 5'-strand, respectively. (**D**) High-throughput shRNA cleavage assays for six groups of randomized shRNAs were conducted with DICER-WT and DICERΔdsRBD. (**E**) A cloning strategy was employed for randomized shRNA substrates and the products cleaved by DICER. (**F**) The overhang length fraction of double-cleavage fragments produced by DICER-WT and DICERΔdsRBD, as detected in sequencing libraries. (**G**) The DC/SC ratios of DICER-WT and DICERΔdsRBD from the high-throughput cleavage assays are presented in cumulative plots, respectively. (**H**) The DC/SC ratios of DICER-WT and DICERΔdsRBD from the cleavage assays depicted in Figure 1J.

Supplementary Figure S2

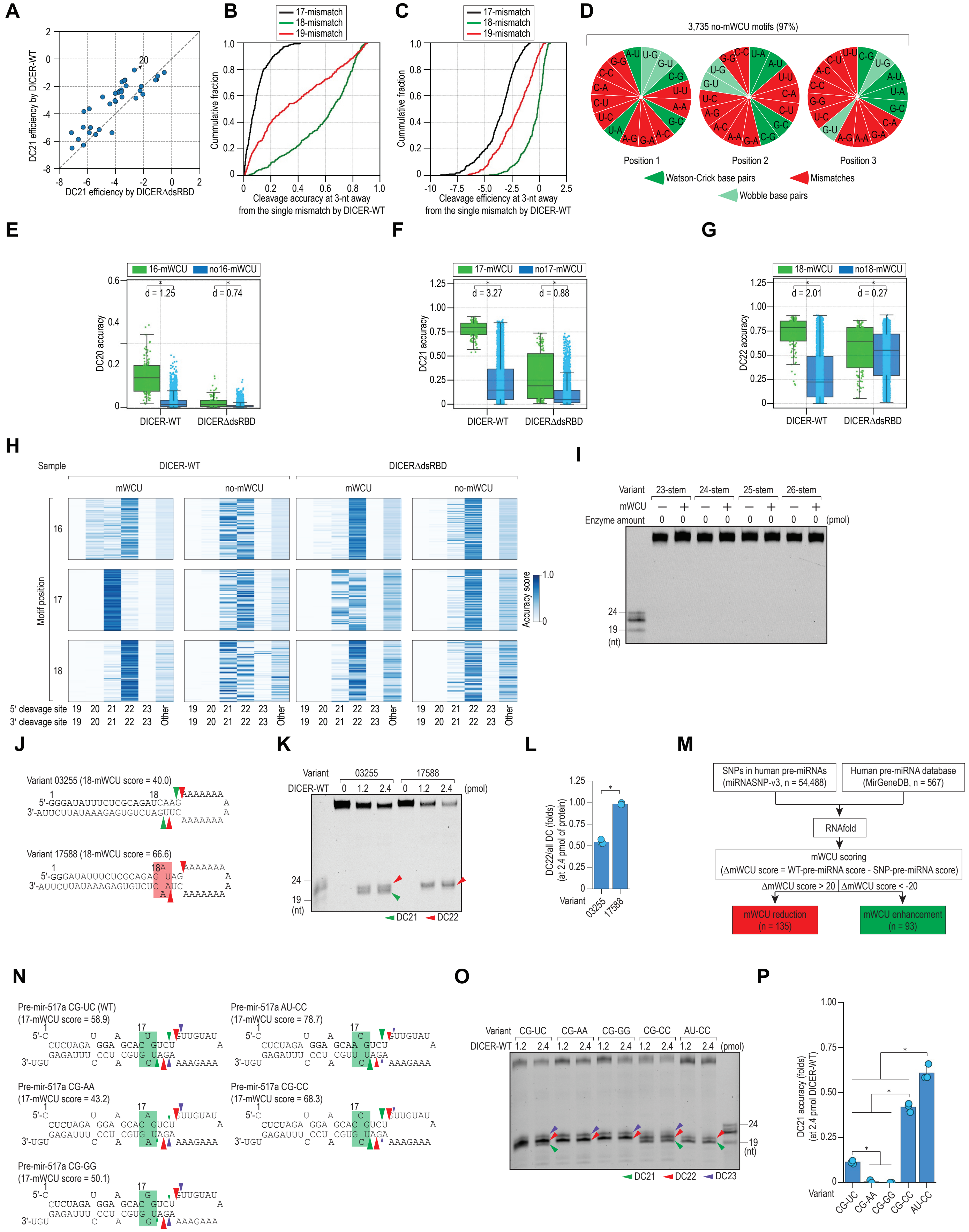

Supplementary Figure S2. mWCU enhances the DICER cleavage via dsRBD.

(**A**) A scatter plot compares the DC21 efficiency of DICER-WT and DICERΔdsRBD in 36 shRNA structures. (**B**, **C**) Cumulative plots show the comparison of cleavage accuracy (**B**) and cleavage efficiency (**C**) at the position 3 nt away from the single mismatch. (**D**) The nt composition of the no-mWCU motifs. Each position contained 16 possible combinations of two nt on the 5'- and 3'-strands. The first and second nt in the combination, for example, A-U, are on the 5'- and 3'-strands, respectively. (**E–G**) The DC cleavage accuracy scores of DICER and DICERΔdsRBD were compared between the mWCU and no-mWCU shRNAs. The numbers in 16-mWCU, 17-mWCU, and 18-mWCU indicate the position of W in shRNAs. Each dot represents one variant harboring the indicated motif. *: $p < 0.05$. (**H**) Cleavage accuracy score by DICER-WT and DICERΔdsRBD for each double cleavage site from position 19 to 23 in shRNAs with or without mWCU motif in positions 16, 17, and 18. One line represents one shRNA variant. (**I**) Gel image of shRNA variants shown in Figure 2O without the addition of enzyme. (**J**) The structures and sequences of shRNA substrates, with green and red arrowheads indicating DC21 and DC22 cleavages, respectively. (**K**) *In vitro* cleavage assays of shRNAs in (**J**) with DICER-WT were conducted. (**L**) The ratio of DC22 to all DC was calculated for DICER-WT from three repeated assays conducted in (**K**). *: $p < 0.05$. (**M**) A schematic is provided for the analysis of single nucleotide polymorphisms (SNPs) that modify the mWCU motifs in human pre-miRNAs. Pre-miRNA sequences were sourced from MirGeneDB (1). The list of SNPs found in human pre-miRNAs was procured from miRNASNP-v3 (2). (**N**) The structures and sequences of pre-mir-517a variants containing various mWCU motifs with different scores. (**O**) *In vitro* cleavage assays of pre-miRNAs in (**N**) with DICER-WT were conducted. (**P**) The ratio of DC21 to all DC was calculated from three repeated assays in (**O**).

Supplementary Figure S3

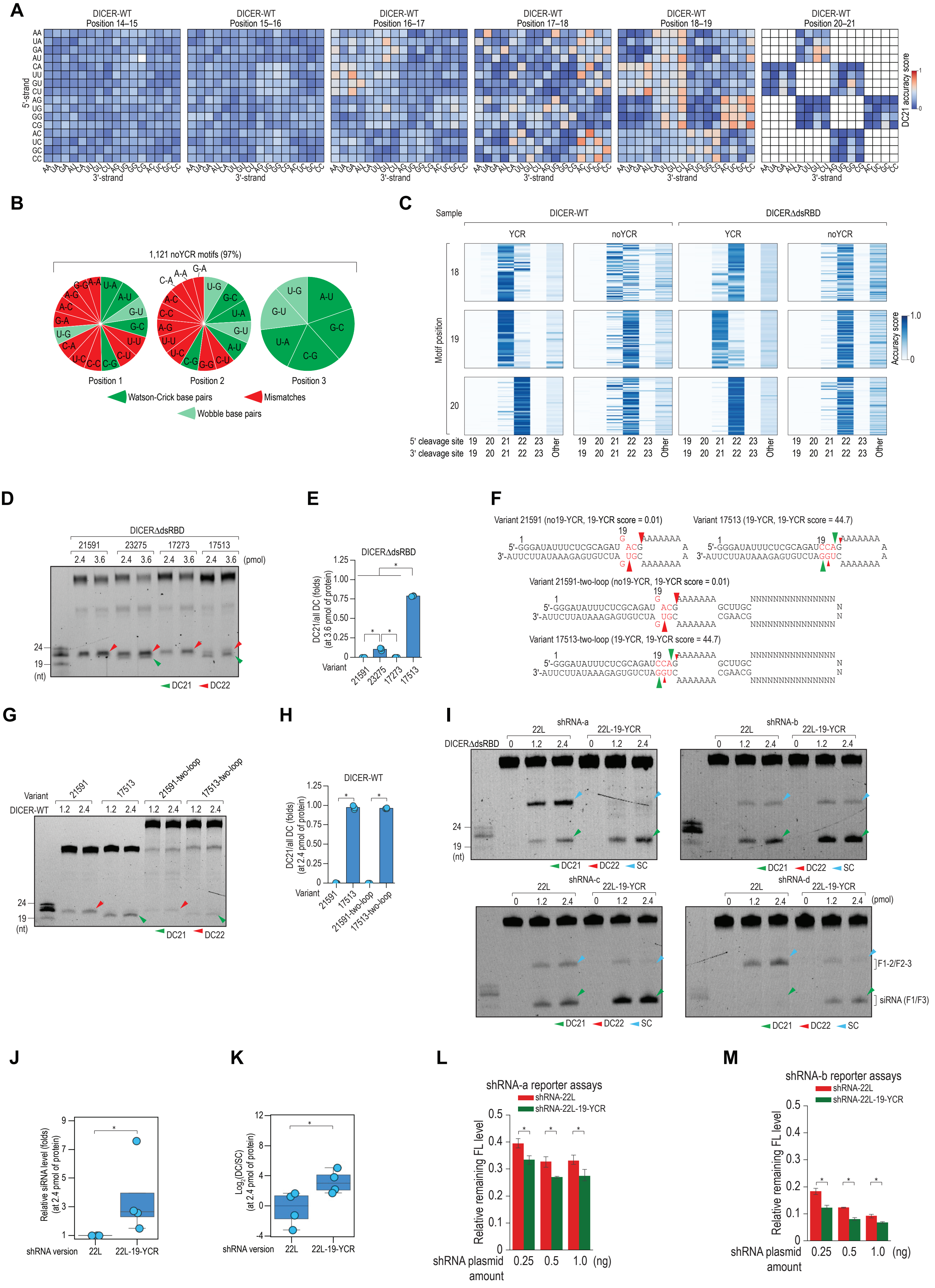

Supplementary Figure S3. The YCR determines cleavage sites of DICER.

(**A**) The DC21 accuracy scores of DICER-WT for dinucleotide combinations in positions 14–15, 15–16, 16–17, 17–18, 18–19, and 20–21 of shRNAs are presented. (**B**) The nt composition of the noYCR motifs. Each position contained 16 possible combinations of two nt on the 5p- and 3p-strands. The first and second nt in the combination, for example, A-U, are on the 5'- and 3'-strands, respectively. (**C**) Cleavage accuracy scores by DICER-WT and DICERΔdsRBD for each double cleavage site from position 19 to 23 in shRNAs with or without YCR motif in position 18, 19, or 20. One line represents one shRNA variant. (**D**) *In vitro* cleavage assays with DICERΔdsRBD for shRNA variants shown in Figure 3H. (**E**) Quantification of DC21 accuracy to all DC for the three repeated cleavage assays in (**D**). (**F**) The sequences and diagrams of one-loop and two-loop shRNAs containing YCR in 19–21 (19-YCR) with varying scores. The green and red arrowheads indicate the DC21 and DC22 cleavages, respectively. (**G**) *In vitro* DICER cleavage assays for shRNA variants shown in (**F**). (**H**) Ratios of DC21/all DC were calculated from three repeated assays shown in (**E**). (**I**) *In vitro* cleavage assays for shRNA variants shown in Figure 3Q using DICERΔdsRBD. SC: single cleavage products. (**J, K**) Relative siRNA level and $\log_2$(DC/SC) of the cleaved products were calculated for DICERΔdsRBD from cleavage assays conducted in (**I**). One dot represents one shRNA variant. *: $p < 0.05$. (**L, M**) The reporter assays of gene "a" (**K**) and gene "b" (**L**) were conducted using the shRNAs shown in Figure 3P. *: $p < 0.05$.

Supplementary Figure S4

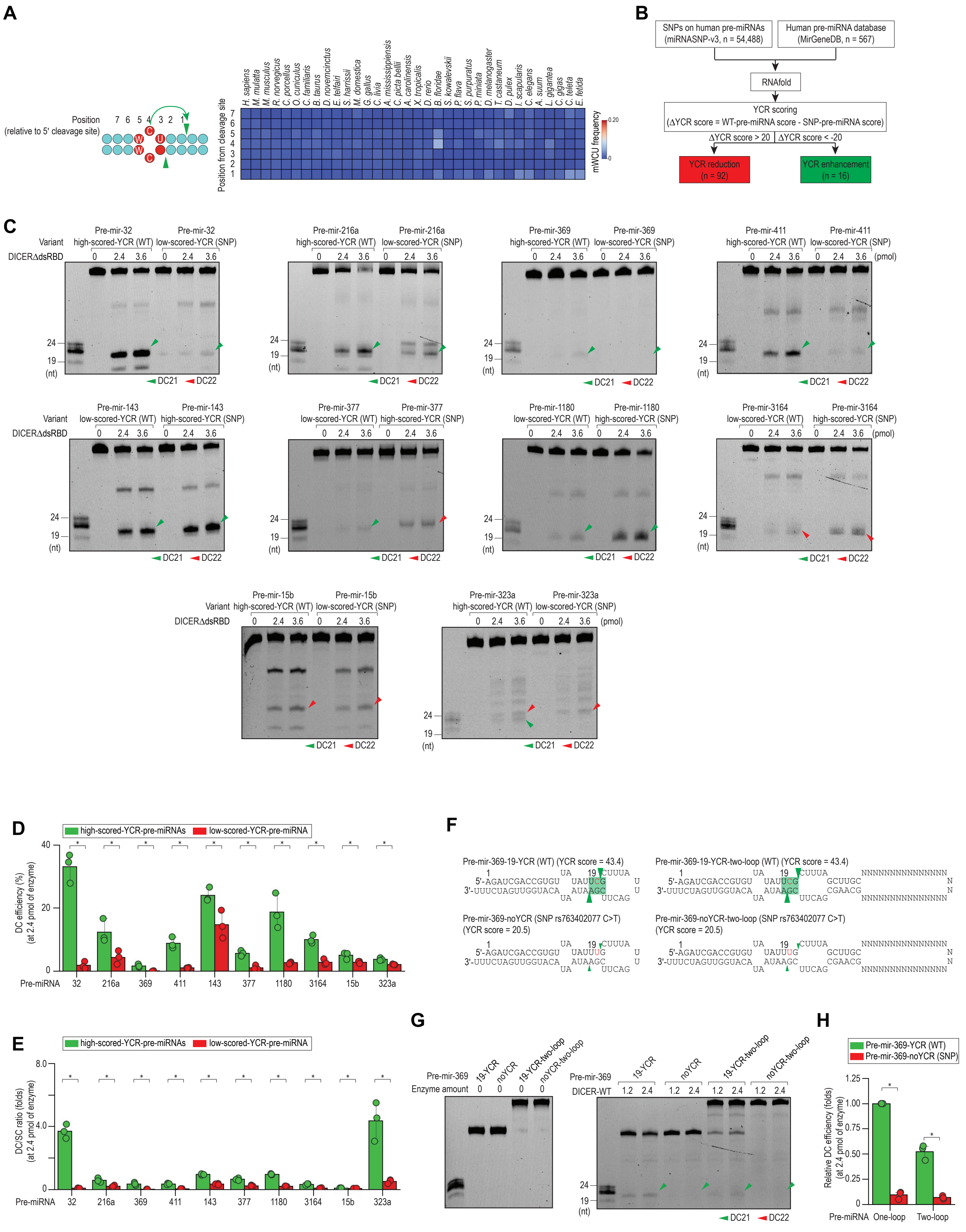

Supplementary Figure S4. Validate functions of YCR in determining cleavage sites of DICER.
(**A**) The enrichment of mWCU motifs is presented. The mWCU frequency was calculated as the ratio of pre-miRNAs containing this motif to all pre-miRNAs. Red circles indicate mWCU motifs. (**B**) A schematic is presented for the analysis of SNPs that alter the YCR motifs in human pre-miRNAs. Pre-miRNA sequences were obtained from MirGeneDB (1). The list of SNPs occurring in human pre-miRNAs was obtained from miRNASNP-v3 (2). (**C**) *In vitro* cleavage assays for pre-miRNAs shown in Figure 4B using DICERΔdsRBD. (**D, E**) The DC cleavage efficiency (**D**) and ratios of DC/SC (**E**) of cleaved products calculated for DICERΔdsRBD from three repeated assays conducted in (**C**). The DC cleavage efficiency was measured as a ratio of the DC product to the pre-miRNA. *: $p < 0.05$. (**F**) The sequences and diagrams of one-loop and two-loop pre-mir-369 containing YCR in 19–21 (19-YCR) with varying scores. The green and red arrowheads indicate the DC21 and DC22 cleavages, respectively. (**G**) *In vitro* DICER cleavage assays for pre-mir-369 variants shown in (**F**). (**H**) The DC efficiency was calculated from three repeated assays in (**G**).

Supplementary Figure S5

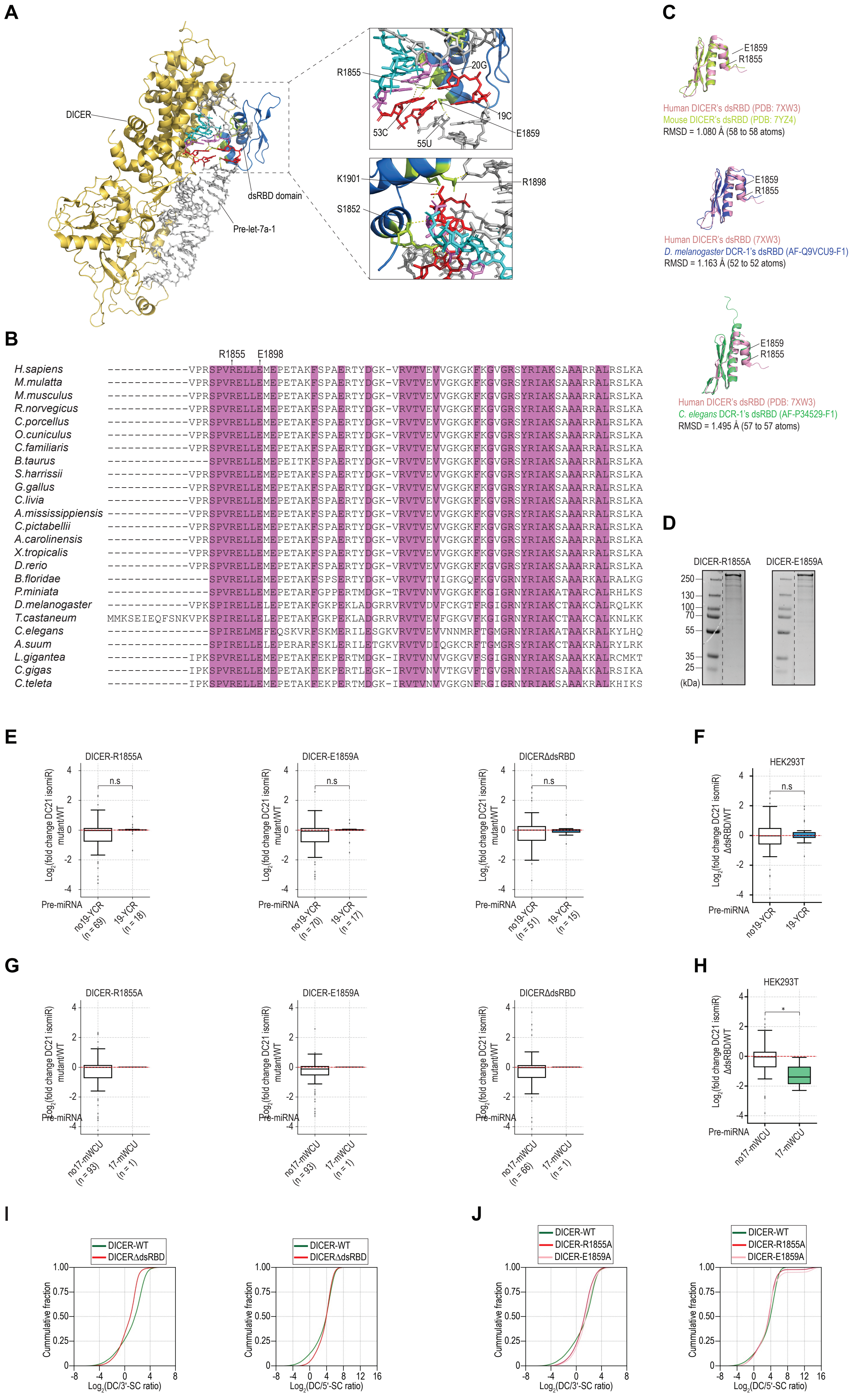

Supplementary Figure S5. Molecular basis of motif recognition.
(**A**) A structure analysis of the pre-let-7a-1 mutant, featuring 18-mWCU and 20-YCR motifs in the dicing state with the human DICER protein, is presented. mWCU and YCR are highlighted in red and cyan, respectively, while the overlapping pair of the two motifs (20 G-C) is depicted in violet. Hydrogen bond prediction was executed using PyMOL (http://www.pymol.org), utilizing the default settings. (**B**) A sequence alignment of the DICER's dsRBD domain from various organisms is provided. The sequences were procured from Uniprot (https://www.uniprot.org/), and the alignment was performed using Multalin (3). A pink color represents residues with a 100% conservation score. (**C**) This panel shows the superimposition of the DICER's dsRBD from the resolved human DICER structures (PDB: 7XW3 and 7YZ4, respectively), alongside the AlphaFold-predicted *D. melanogaster* and *C. elegans* DICER structures (ID: AF-Q9VCU9-F1 and AF-P34529-F1, respectively). The root-mean-square deviation (RMSD) and the number of aligned residues were examined using PyMOL (http://www.pymol.org). (**D**) An evaluation of the purity of DICER-R1855A and DICER-E1859A in SDS-PAGE. (**E, G**) Alterations in the DC21 isomir levels of pre-miRNAs with 19-YCR motifs (**E**) or with 17-mWCU motifs (**G**) in HCT116 DICER-KO cells rescued with DICER-R1855A, DICER-E1859A, or DICER ΔdsRBD. n.s: not significant, *: $p < 0.05$. (**F, H**) Changes in the DC21 isomir levels of pre-miRNAs with 19-YCR motifs (**F**) or with 17-mWCU motifs (**H**) in HEK293T DICER-KO cells rescued with DICERΔdsRBD (4). n.s: not significant, *: $p < 0.05$. (**I–J**) The DC/3'-SC and DC/5'-SC ratios, derived from high-throughput cleavage assays, were compared between DICER-WT and DICER ΔdsRBD (**I**), and between DICER-WT and DICER-R1855A, DICER-E1859A (**J**).

Supplementary Figure S6

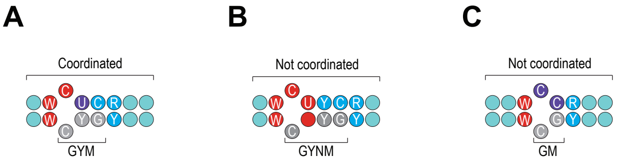

Supplementary Figure S6. Two-motif model for explaining DICER's cleavage site selection.
The reported GYM motif (4) can be considered as a part of the combined mWCU and YCR motifs when they are in the coordinated mode (**A**). When the two motifs are in an incoordinated mode and form a 6-bp length combination, GY and M are separated by one nucleotide (**B**). In another scenario when mWCU and YCR are incoordinated and form a 4-bp length motif, the Y and M of GYM are merged (**C**). Red circles indicate mWCU motifs, blue circles indicate YCR motifs, and purple circles indicate the shared positions between the two motifs. Components of the GYM motif are colored in grey.

Supplementary references

1. Fromm, B., Hoye, E., Domanska, D., Zhong, X., Aparicio-Puerta, E., Ovchinnikov, V., Umu, S.U., Chabot, P.J., Kang, W., Aslanzadeh, M. et al. (2022) MirGeneDB 2.1: toward a complete sampling of all major animal phyla. *Nucleic Acids Research*, **50**, D204-D210.
2. Liu, C.-J., Fu, X., Xia, M., Zhang, Q., Gu, Z. and Guo, A.-Y. (2021) miRNASNP-v3: a comprehensive database for SNPs and disease-related variations in miRNAs and miRNA targets. *Nucleic Acids Research*, **49**, D1276-D1281.
3. Corpet, F. (1988) Multiple sequence alignment with hierarchical clustering. *Nucleic Acids Research*, **16**, 10881-10890.
4. Lee, Y.Y., Kim, H. and Kim, V.N. (2023) Sequence determinant of small RNA production by DICER. *Nature*.
